# Re-directing bacterial microcompartment systems to enhance recombinant expression of lysis protein E from bacteriophage ϕX174 in *Escherichia coli*

**DOI:** 10.1186/s12934-017-0685-x

**Published:** 2017-04-26

**Authors:** Mimi C. Yung, Feliza A. Bourguet, Timothy S. Carpenter, Matthew A. Coleman

**Affiliations:** Biosciences and Biotechnology Division, Physical and Life Sciences Directorate, Lawrence Livermore National Laboratory, 7000 East Avenue, L-452, Livermore, CA 94550 USA

**Keywords:** Bacterial microcompartment, BMC, Lysis protein E, Bacteriophage phiX174, Toxic protein expression

## Abstract

**Background:**

Recombinant expression of toxic proteins remains a challenging problem. One potential method to shield toxicity and thus improve expression of these proteins is to encapsulate them within protein compartments to sequester them away from their targets. Many bacteria naturally produce so-called bacterial microcompartments (BMCs) in which enzymes comprising a biosynthetic pathway are encapsulated in a proteinaeous shell, which is in part thought to shield the cells from the toxicity of reaction intermediates. As a proof-of-concept, we attempted to encapsulate toxic, lysis protein E (E) from bacteriophage ϕX174 inside recombinant BMCs to enhance its expression and achieve higher yields during downstream purification.

**Results:**

E was fused with various N-terminal BMC targeting tags (PduP-, PduD-, and EutC-tags, 18–20 amino acids) and co-expressed with appropriate BMC shell proteins that associate with the tags and are required to form BMCs. Only BMC targeted E fusions, but not non-tagged E, could be successfully cloned, suggesting that the BMC tags reduce the toxicity of E. A PduP-tagged E system appeared to achieve the highest expression of E. Co-expression of Pdu BMC shell proteins with PduP-E increased its expression by 20–50%. Affinity purification of PduP-E via Ni–NTA in the presence of Empigen BB detergent yielded 270 µg of PduP-E per L of induced culture. Removal of the PduP-tag via proteolysis resulted in a final yield of 200 µg of E per L of induced culture, a nearly order of magnitude (~sevenfold) improvement compared to prior reports.

**Conclusions:**

These results demonstrate improved expression of ϕX174 lysis protein E via re-directed BMC systems and ultimately higher E purification yields. Similar strategies can be used to enhance expression of other toxic proteins in recombinant *Escherichia coli* systems.

**Electronic supplementary material:**

The online version of this article (doi:10.1186/s12934-017-0685-x) contains supplementary material, which is available to authorized users.

## Background

Heterologous expression of proteins in host organisms plays a central role in biochemistry and synthetic biology. Protein expression, however, can be challenging, especially if the protein of interest is toxic to the host organism [1, 2]. Several methods have been developed to overcome toxicity during protein expression, including use of tightly-regulated promoters and fusion proteins that shield toxicity [3]. Another potential method to overcome toxicity is to encapsulate the toxic protein within other shell proteins, in order to sequester it from interactions in the host organism that would lead to its toxicity [4]. Many bacteria have evolved a mechanism to compartmentalize proteins comprising a biosynthetic pathway inside self-assembling protein shells, called bacterial microcompartments (BMCs) [5]. BMCs are thought to prevent toxicity of reaction intermediates in the biosynthetic pathway, as well as increase the reaction efficiency of the pathways by increasing the local concentration of enzymes and substrates within the compartments. Re-directing BMC shell proteins to encapsulate toxic proteins, thus, has the potential to shield the toxicity of recombinant proteins, enabling downstream applications such as purification and enhanced activity for a desired function.

The most studied examples of BMCs in Nature are carboxysomes involved in carbon fixation, and 1,2-propanediol utilization (Pdu) and ethanolamine utilization (Eut) pathways found in enteric bacteria [5]. Several recent studies have identified components to successfully re-direct non-native proteins to the interior of Pdu and Eut BMCs, including the genes responsible for forming empty BMCs as well as short N-terminal α-helical sequences that interact with BMC shells proteins for interior targeting, presumably through hydrophobic interactions [6, 7]. Expression of *pduABJKNU* from *Citrobacter freundii* was found to be sufficient for production of empty Pdu BMCs [8], while *eutSMNLK* and *eutS* alone from *Salmonella enterica* were found to produce empty Eut BMCs [9]. Other studies showed that GFP could be targeted to the interior of Pdu BMCs by tagging the N-terminus of GFP with the N-terminal 18–20 amino acids of interior proteins PduP or PduD in the Pdu degradation pathway [10–12]. Similarly, for the Eut pathway, fusing the N-terminal 19 amino acids of EutC or very recently, the N-terminal 20 amino acids of EutE to the N-terminus of GFP targeted it to Eut BMCs [9, 13, 14]. These studies provide a toolkit for encapsulating toxic proteins inside BMCs.

As a proof-of-concept, we attempted to encapsulate lysis protein E from bacteriophage ϕX174 (herein abbreviated as E) inside recombinant BMCs in *Escherichia coli* to improve expression and downstream purification of this toxic protein. E is a 91-residue membrane protein that is responsible for bacterial cell lysis by ϕX174 phage and thus is extremely toxic to *E. coli*. E blocks host cell wall biosynthesis by inhibiting the membrane-bound translocase MraY, which catalyzes the formation of Lipid I [15]. Over-expression of E in *E. coli* has been difficult, as it induces rapid cell death within 15 min [16]. Young and coworkers have succeeded in over-expressing and purifying E from *E. coli* by co-expressing E with MraY from *Bacillus subtilis*, which is relatively insensitive to E, to partially prevent lysis of the *E. coli* host. Ultimately, they purified 27 µg of E per L of culture to 84% purity [16]. Here, E fused to different N-terminal BMC targeting tags was co-expressed with appropriate BMC shell proteins. We report that addition of N-terminal targeting tags onto E allows it to be expressed at relatively high levels (~6–8 mg/L of induced culture with PduP-tag), while co-expression of BMC shell proteins with tagged E can increase expression by 20–50% in a PduP-tagged/Pdu BMC system. Purification by Ni–NTA chromatography in the presence of Empigen BB (EBB) detergent yielded 270 µg of PduP-E and 200 µg of E (after cleavage of the PduP-tag) per L of induced culture (~80% pure), a ~sevenfold improvement in yield compared to prior reports [16].

## Methods

### Materials

All chemicals were purchased from Sigma-Aldrich (St. Louis, MO, USA) unless otherwise noted. Lysogeny broth (LB) and agar were purchased from Amresco (Solon, OH, USA). Where applicable, LB was supplemented with antibiotics at the following concentrations in both solid and liquid media: 50 µg/mL for kanamycin and 100 µg/mL for carbenicillin or ampicillin. All PCR reactions were amplified using iProof polymerase from Bio-Rad (Hercules, CA, USA). All restriction enzymes and competent *E. coli* cells were purchased from New England Biolabs (NEB, Ipswich, MA, USA). The sequences of all plasmids were confirmed by DNA sequencing (Elim, Newark, CA, USA). All plasmids and bacterial strains used in this study are listed in Table 1 and all primers in Additional file 1: Table S1. *Bacillus licheniformis* (ATCC 14580) was purchased from the American Type Culture Collection (ATCC, Manassas, VA, USA).

**Table 1:** Plasmids and bacterial strains used in this study

Plasmid/strain	Description	Reference
Plasmids		
pD444-SR	Expression vector, IPTG-inducible T5 promoter, *lacI*, *ori pUC*, *amp* (Am^r^)	ATUM
pD861	Expression vector, rhamnose-inducible *pRha* promoter, *ori pUC*, *kan* (Km^r^)	ATUM
pMCY29	pD444-SR-derived vector expressing *eutSMNLK*	This study
pMCY30	pD444-SR-derived vector expressing *pduABJKNU*	This study
pMCY31	pD444-SR-derived vector expressing *eutS*	This study
pMCY85	pD861-derived vector expressing the gene for EutC^1–19^-X-E-T-His_6_ fusion	This study
pMCY86	pD861-derived vector expressing the gene for PduD^1–20^-X-E-T-His_6_ fusion	This study
pMCY87	pD861-derived vector expressing the gene for PduP^1–18^-X-E-T-His_6_ fusion	This study
pMCY90	pD861-derived vector expressing the gene for PduP^1–18^-X-mCherry-T-His_6_ fusion	This study
pMCY99	pD444-SR-derived vector expressing *mcherry* and *pduJKNU*	This study
pMCY101	pD444-SR-derived vector expressing *mcherry*	This study
Strains		
*E. coli* BL21	Expression strain for non-T7 protein expression, *fhuA2 [lon] ompT gal [dcm]* Δ*hsdS*	NEB
Ec0030	*E. coli* BL21 harboring pMCY30	This study
Ec2985	*E. coli* BL21 harboring pMCY29 and pMCY85	This study
Ec3185	*E. coli* BL21 harboring pMCY31 and pMCY85	This study
Ec3086	*E. coli* BL21 harboring pMCY30 and pMCY86	This study
Ec0087	*E. coli* BL21 harboring pMCY87	This study
Ec3087	*E. coli* BL21 harboring pMCY30 and pMCY87	This study
Ec3090	*E. coli* BL21 harboring pMCY30 and pMCY90	This study
Ec9987	*E. coli* BL21 harboring pMCY99 and pMCY87	This study
Ec10187	*E. coli* BL21 harboring pMCY101 and pMCY87	This study

*X* factor Xa protease cleavage site, *T* thrombin protease cleavage site, *E* lysis protein E

### Design of BMC plasmids pMCY29, pMCY30, and pMCY31

Plasmids pMCY29 and pMCY30 were synthesized and purchased from ATUM (formerly DNA2.0, Newark, CA, USA). Plasmid pMCY29 contains gene cassettes for *eutS*, *eutMN*, and *eutLK* from the *S. enterica* LT2 genome (NC_003197.1), placed in tandem, under the control of the *T5* promoter in pD444-SR (ATUM). The following nucleotide linker regions containing ribosomal re-initiation sites were placed between *eutS* and *eutMN* and between *eutMN* and *eutLK*, respectively: linker 1 (5′CTAGGAATAATTTTGTTTAACTTTAAGAAGGAGATAAAAA3′) and linker 2 (5′CAAGCGACTTAAATCAATATCTTTAAGAAGGAGATAAAAA3′). A *Spe*I site and a *Nhe*I site were placed immediately downstream of the *eutS* gene and the *eutMN* cassette, respectively. To obtain pMCY31, pMCY29 was digested with *Spe*I and *Nhe*I to excise *eutMNLK* and the resulting, truncated vector fragment was re-ligated together using T4 DNA ligase (NEB); *Spe*I and *Nhe*I produce compatible ends for ligation. Plasmid pMCY30 contains gene cassettes for *pduAB*, *pduJK*, *pduN*, and *pduU* from the *S. enterica* LT2 genome, placed in tandem, under the control of the *T5* promoter in pD444-SR. To insert ribosome re-initiation sites, nucleotide linker 1 was placed between *pduAB* and *pduJK* and between *pduN* and *pduU*, while linker 2 was placed between *pduJK* and *pduN*. A *Spe*I site and a *Nhe*I site were placed immediately downstream of the *pduN* and *pduU* genes, respectively, for excision of *pduU* if necessary. Plasmid maps for pMCY29 and pMCY30 can be found in Additional file 1: Figure S1.

### Construction of E-expression plasmids pMCY85, pMCY86, and pMCY87

Plasmids pEutC, pPduD, and pPduP were synthesized and purchased from ATUM. Plasmid pEutC contains the codon-optimized nucleotide sequence (ATUM, [17]) encoding for the following features under the control of the *pRha* promoter in pD481: an N-terminal EutC-tag comprised of the first 19 amino acids of EutC from *S. enterica* LT2 (MDQKQIEEIVRSVMASMGQ), followed by a Factor Xa cleavage site, a thrombin cleavage site, and a C-terminal hexa-histidine (His_6_) tag. A *Bgl*II site was placed between the nucleotide regions encoding the two protease cleavage sites for inserting the desired gene of interest into the plasmid. Plasmids pPduD and pPduP are identical in design to pEutC, except that the codon-optimized nucleotide region encoding the EutC-tag was replaced with that of a PduD-tag (the first 20 amino acids of PduD, MEINEKLLRQIIEDVLRDMK) and a PduP-tag (the first 18 amino acids of PduP, MNTSELETLIRTILSEQL), respectively, both from *S. enterica* LT2. Codon-optimized sequences for pEutC, pPduD, and pPduP can be found in Additional file 1: Table S2.

To construct plasmids pMCY85, pMCY86, and pMCY87, the *E* gene was first PCR amplified from bacteriophage ϕX174 genomic DNA (NEB) using primers *lysE_for* and *lysE-_rev*. The resulting PCR product was ligated into the *Bgl*II site in pEutC, pPduD, and pPduP by two-fragment InFusion reactions (Clontech In-Fusion HD Cloning Plus kit, Mountain View, CA, USA) to construct pEutC-E, pPduD-E, and pPduP-E, respectively. The generated upstream *Bgl*II site (between the nucleotide region for the Factor Xa site and the *E* gene) was removed by site-directed mutagenesis to place the Factor Xa cleavage site directly adjacent to E in the expressed fusion protein. Site-directed mutagenesis was carried out by PCR amplification of pEutC-E, pPduD-E, and pPduP-E with the following primer sets, respectively: *eutCdBgl*II*/dBgl*II*_for*, *pduDdBgl*II*/dBgl*II*_for*, and *pduPdBgl*II*/dBgl*II*_for*. Self-ligation by InFusion reaction of the PCR amplified products generated plasmids pMCY85, pMCY86, and pMCY87, respectively.

### Construction of control plasmids pMCY90, pMCY99, and pMCY101

To obtain pMCY90 expressing PduP-mCherry, the *mcherry* gene was PCR amplified using primers *mcherry_for* and *mcherry_rev* from plasmid pRVCHYC-2 [18]. The pPduP-E vector was also PCR amplified using primers *pPduPlysE_for* and *pPduPlysE_rev*. The PCR fragments were ligated together by InFusion reaction to generate pPduP-E-mCherry, which was then digested with *Bgl*II and self-ligated using T4 DNA ligase to excise the *E* gene and generate pMCY90.

Plasmids pMCY99 and pMCY101 are derivatives of pMCY30 in which the *mcherry* gene replaces the *pduAB* cassette and the entire *pduABJKNU* cassette, respectively. For pMCY99, the *mcherry* gene was PCR amplified from pPduP-E-mCherry using primers *mcherry_for2* and *mcherry_rev2*, containing overlap regions with nucleotides 7-21 in *pduA* and the last 15 nucleotides of *pduB,* respectively. For pMCY101, the *mcherry* gene was PCR amplified from pPduP-E-mCherry using primers *mcherry_for2* and *mcherry_rev3*, which contains an overlap region with the 12 nucleotides immediately downstream of *pduU* in pMCY30. The pMCY30 vector without *pduAB* and without *pduABJKNU* were PCR amplified using primer sets *pduAup_rev/pduBdown_for* and *pduAup_rev/pduUdown_for*, respectively. Appropriate two fragment InFusion reactions were conducted to generate pMCY99 and pMCY101.

### Growth and induction conditions

Carbenicillin was used in place of ampicillin in all cultures. All optical density at 600 nm (OD_600_) measurements were recorded on a Tecan Sunrise plate reader instrument (Männedorf, Switzerland) with 200 µL of culture in a standard 96-well plate.

For E expression experiments, *E. coli* cells were pre-cultured from single colonies in 5 mL of LB supplemented with appropriate antibiotic(s) at 25 °C for 16 h. Cells were then diluted into 10 mL of LB supplemented with appropriate antibiotic(s) to an initial OD_600_ of 0.03 in a 50-mL flask and were cultured at 30 °C and 220 rpm. Isopropyl β-d-1-thiogalactopyranoside (IPTG) was added to the cultures to a final concentration of 0.5 mM at the start of growth (t = −3 h) or once the cultures reached an OD_600_ of ~0.4 (t = 0) where indicated. Rhamnose was added to the cultures to final concentrations of 0.1, 0.2, or 0.5 mM once the cultures reached an OD_600_ of ~0.4.

For isolation of BMCs and purification of PduP-E, *E. coli* cells were pre-cultured from single colonies in 20 mL of LB supplemented with appropriate antibiotic(s) in a 250-mL flask at 25 °C for 16 h. Cells were then diluted into 200 mL of LB supplemented with appropriate antibiotic(s) to an initial OD_600_ of 0.03 in a 1-L flask. Cells were cultured at 30 °C and 220 rpm and harvested at 3 h post-rhamnose induction. All induction conditions with IPTG and rhamnose are the same as above.

### Whole cell SDS-PAGE and Western blot

At the indicated post-rhamnose induction time points, cells from 500 µL of culture were harvested at 16,000×*g* for 2 min and immediately re-suspended in 100 µL of 1× Laemmli buffer. Samples were boiled for 10 min at 100 °C, cooled to room temperature, and stored at −80 °C until analysis. For SDS-PAGE analysis, samples were resolved using 18% Criterion TGX or any-kDa Mini-Protean TGX gels (Bio-Rad, Hercules, CA, USA) and stained with Coomassie blue stain. For Western blot analysis, appropriate dilutions of the samples were resolved on an 18% Criterion TGX gel. Protein was blotted to a PVDF membrane using the Bio-Rad Transblot Turbo system at 2.5 A for 7 min (up to 25 V, Mixed Mw protocol). Membranes were blocked in blocking buffer containing 3% (w/v) milk and 0.1% (v/v) Tween20 in TBS buffer (20 mM Tris, pH 7.4, 150 mM NaCl). PduP-E protein was probed using mouse monoclonal anti-histidine tag (anti-His_6_, Clone AD1.1.10, Bio-Rad antibodies) at 1 µg/mL in blocking buffer and goat anti-mouse IgG1-horseradish peroxidase conjugate (Bio-Rad antibodies) at a 1/5000 dilution in blocking buffer. Protein was detected by chemiluminescence using SuperSignal West Pico chemiluminescent substrate (ThermoFisher, Waltham, MA, USA). To generate a PduP-E standard curve for Western blot, 3–34 ng of purified PduP-E were spiked with Ec0030 (*E. coli* BL21 harboring pMCY30) cells containing the same OD_600_*µL as the unknown samples in Laemmli buffer. Ec0030 cells were provided as cellular background to ensure blotting consistency with unknown samples. The concentration of PduP-E was determined by absorbance at 280 nm (ε = 19,990/M cm [19]).

### Isolation of BMCs

BMCs were isolated from Ec0087, Ec3087, and Ec3090 cells following the procedure described by Sinha et al. [20], with minor modifications. Cell pellet (~0.7–1.0 g) was re-suspended in ~4 mL of 60% BPER-II (ThermoFisher) in buffer A (50 mM Tris, pH 8.0, 500 mM KCl, 12.5 mM MgSO_4_), supplemented with 0.1 mg/mL lysozyme, 1 mM phenylmethylsulfonyl fluoride (PMSF), and 10 U/mL DNaseI. Cells were lysed via a French pressure cell at 12,000 psi and centrifuged at 12,000×*g* for 10 min at 4 °C to remove cell debris. The soluble fraction was then centrifuged at 20,000×*g* for 20 min at 4 °C to collect the BMCs. The BMCs were washed with 1 mL of 60% BPER-II in buffer A and then harvested again at 20,000×*g* for 20 min at 4 °C. Crude BMCs were re-suspended in 400 µL of buffer B (50 mM Tris, pH 8.0, 50 mM KCl, 5 mM MgCl_2_). The BMC suspension was then centrifuged at 12,000×*g* for 1 min at 4 °C three times to remove insoluble contaminants. The final purified BMC suspension was stored at 4 °C until use. Total protein content was determined by Bradford assay (Bio-Rad) using BSA as a standard [21].

### Transmission electron microscopy (TEM) sample preparation and analysis

Purified BMC suspension, 10 µL, was added to a 200-mesh, carbon formvar-coated copper grid and incubated at room temperature for 2 min. Excess liquid was removed using filter paper. The grid was then stained with 10 µL of 2% uranyl acetate for 1 min at room temperature, after which excess liquid was again removed using filter paper. The grid was dried and stored at room temperature until TEM analysis. TEM analysis was performed on a Titan transmission electron microscope (FEI, Hillsboro, OR, USA).

### Purification of E from whole cells and isolated BMCs

For purification from whole cells, ~0.7–1.0 g of wet cell paste was re-suspended in ~4 mL of Tris buffer (100 mM Tris, pH 8.0, 100 mM NaCl) supplemented with 10 U/mL DNaseI. Cells were lysed via a French pressure cell at 12,000 psi. Cellular lysates were centrifuged at 5000×*g* at 4 °C for 10 min to remove unbroken cells. EBB detergent (ThermoFisher) was added to the supernatant to a final concentration of 2% and rocked at 4 °C overnight. The supernatant was then centrifuged at 20,000×*g* at 4 °C for 25 min to remove insoluble cellular debris. The clarified supernatant was diluted by fivefold using 20 mM imidazole in Tris buffer to a final EBB concentration of 0.4% and added to 250 µL of Ni–NTA resin (Qiagen, Hilden, Germany) pre-equilibrated with Tris buffer. Protein and resin were rocked at 4 °C for 1 h, after which the resin was packed into a column and the flow through was collected. The column was washed with 8 × 0.5 mL of 20 mM imidazole, 0.06% EBB in Tris buffer and eluted with 10 × 180 µL of 200 mM imidazole, 0.06% EBB in Tris buffer. The two most concentrated E fractions (elution fractions E4-E5) were pooled and dialyzed against 0.06% EBB in Tris buffer using a Slide-A-Lyzer dialysis device with a molecular weight cutoff of 3.5 kDa to remove the imidazole. Purified protein was quantified by absorbance at 280 nm.

For purification from isolated BMCs, EBB detergent was added to 660 µL of purified BMC suspension (2.4 mg/mL total protein) to a final concentration of 2%. The sample was rocked at 4 °C for 2 h to solubilize the PduP-E protein, then diluted by fivefold using 20 mM imidazole in Tris buffer to a final EBB concentration of 0.4%, and added to 100 µL of Ni–NTA resin pre-equilibrated with Tris buffer. Protein and resin were rocked at 4 °C for 30 min. The resin was packed into a column and the flow through was collected. The column was washed with 8 × 200 µL of 20 mM imidazole, 0.06% EBB in Tris buffer and eluted with 10 × 75 µL of 200 mM imidazole, 0.06% EBB in Tris buffer. Fractions were analyzed by SDS-PAGE and Western blot.

### Factor Xa cleavage of PduP-E and purification of non-tagged E

Purified PduP-E, 7.3 µg, was treated with 0.5 µg of Factor Xa protease (NEB) in 50 µL of 0.06% EBB in Tris buffer for 6 h at 23 °C. The reaction was then added to 3 µL of Ni–NTA resin and rocked at room temperature for 30 min to partially remove the ~25 kDa contaminant. Ni–NTA resin was removed by centrifugation at 1000×*g* and protein was recovered. PMSF was added to 0.1 mM and incubated at room temperature for 15 min to inactivate the protease. The mixture was then directly used in growth inhibition assays.

### *Bacillus licheniformis* growth inhibition assays with purified PduP-E or non-tagged E

Assays were conducted per methods described in Mendel et al. [22]. *B. licheniformis* cells were pre-cultured from single colonies in 5 mL of LB at 30 °C for 16 h. Cells were diluted to an initial OD_600_ of 0.005 and 100 µL aliquots were dispensed in a 96-well plate. Purified PduP-E or non-tagged E was added to culture to 0.5 µM using 10 µM protein stocks containing 0.06% EBB in Tris buffer. As a buffer control, an equal volume of 0.06% EBB in Tris buffer was added to culture. Cultures were grown in a PHMP-4 Microplate Shaker (Grant Instruments, Cambridge, England) at 37 °C and 1000 rpm. Growths were monitored by OD_600_. Activity was measured as the percent growth inhibition after 6 h of growth compared to normal growth based on OD_600_.

## Results

### Design of different BMC systems to support expression of E

**Fig. 1:**
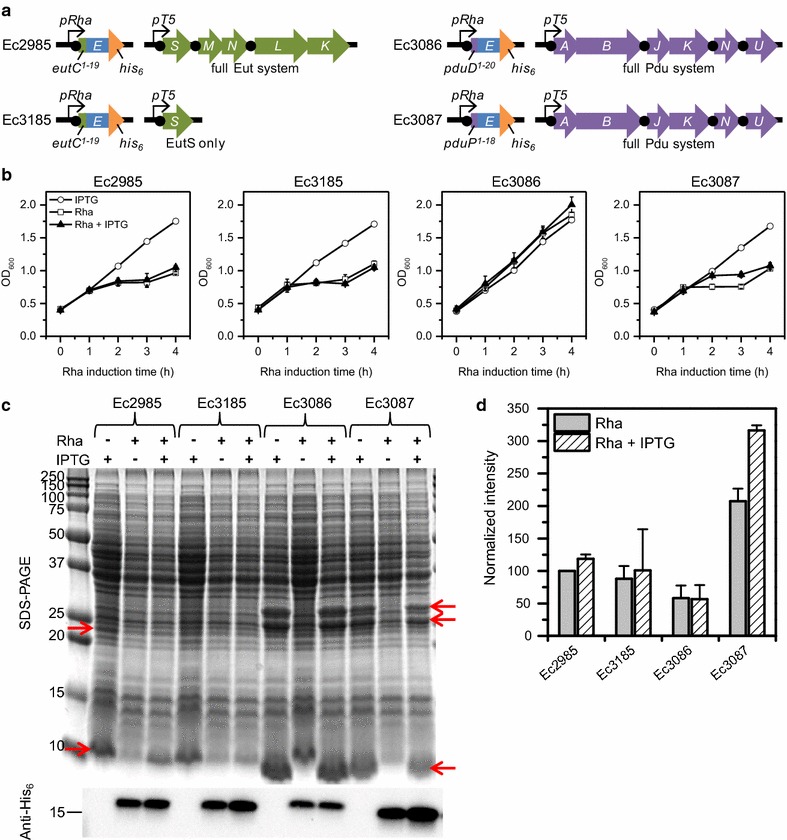
Growth and E expression in different BMC systems. **a** Genetic scheme for the different BMC systems. *Black circles* represent ribosome binding/re-initiation sites. **b** Growth curves for each strain under different induction conditions. Induction with 0.5 mM IPTG only at t = −3 h (*open circle*); induction with 0.1 mM rhamnose only at t = 0 (*open square*); and co-induction with both IPTG and rhamnose (*filled triangle*). *Error bars* represent data from 3 replicates. **c** SDS-PAGE and anti-His_6_ Western blot at 4 h post-rhamnose induction from 1 representative growth. The equivalent of 50 µL and 2 µL of original culture were analyzed by SDS-PAGE and Western blot, respectively. *Left red arrows* EutL (*top*), EutS/M/N (*bottom*). *Right red arrows* PduB (*top*), PduB′ (*middle*), and PduA/J (*bottom*). Sizes of molecular weight standards in kDa are shown on the *left*. **d** Relative Western blot intensities compared to Ec2985, Rha only. *Gray bars* rhamnose only induction; *striped bars* IPTG and rhamnose co-induction. *Error bars* represent data from 3 replicates from the different growths in **b** (see Additional file 1: Figure S2 for raw data)

In order to target E to the interior of BMCs, E was expressed with known N-terminal BMC targeting tags (18–20 amino acids) in combination with appropriate BMC shell proteins from Eut and Pdu operons in *S. enterica*, which were previously shown to produce empty, recombinant BMCs [8–10, 12]. *E. coli* strains were constructed to express the following: *eutC*-*E* and *eutSMNLK* (Ec2985); *eutC*-*E* and *eutS* (Ec3185); *pduD*-*E* and *pduABJKNU* (Ec3086); and *pduP*-*E* and *pduABJKNU* (Ec3087) (Fig. 1a). The gene for each E construct was expressed from a rhamnose inducible promoter (*pRha*), which was chosen for tunable expression, in pD861 with a kanamycin resistance marker. The *pRha* promoter was also chosen over arabinose and IPTG inducible promoters because it has been shown to be more rheostatic, in that expression levels per cell correlated with inducer concentration, rather than producing an all-or-none response [23]. This is an important feature when modulating the stoichiometry between the E constructs and BMC shell proteins. The BMC shell proteins were expressed from a T5 IPTG inducible promoter in pD444-SR with an ampicillin resistance marker. All E constructs were designed to express a C-terminal His_6_-tag for later purification of the protein. Factor Xa and thrombin protease cleavage sites were engineered appropriately for potential cleavage of the BMC targeting and His_6_ tags, respectively. The factor Xa protease cleavage site was placed directly adjacent to the E protein, so that upon proteolytic cleavage, the native N-terminal E sequence would remain.

### Different BMC systems alter protein expression levels and toxicity of E

To compare E expression, the different *E. coli* strains were grown in LB medium and induced under one of the following conditions: IPTG only (BMC), rhamnose only (E), or rhamnose and IPTG co-induction (E + BMC). IPTG was added to 0.5 mM at the start of growth, while rhamnose was added to 0.1 mM once cells reached an OD_600_ of 0.4, about 3 h after the start of growth. The timing of IPTG induction was initially chosen to be well before rhamnose induction to ensure that enough BMC protein is available for encapsulation once E is expressed. Growths under the different induction conditions for each E/BMC combination strain were monitored by OD_600_ (Fig. 1b) and after 4 h rhamnose induction, equivalent volumes of culture were analyzed by SDS-PAGE and anti-His_6_ Western blot (Fig. 1c, d; Additional file 1: Figure S2).

In all strains, the IPTG only growth curves showed normal growth for *E. coli,* demonstrating that expression of the different BMC shell proteins alone is not toxic to the cells. SDS-PAGE and Western blot analysis of the IPTG only samples confirmed that the appropriate BMC shell proteins (Fig. 1c, red arrows) were expressed and E levels were undetectable. Over-expression of Pdu BMC shell proteins was observed to be higher than that of the Eut BMC shell proteins based on the intensities of the relevant protein bands. In the Pdu strains, clear over-expression of PduB, PduB′, PduA, and PduJ were observed (the *pduB* gene produces two protein products PduB and PduB′, which are ~27 and 25 kDa, respectively [24]). PduK, PduN, and PduU were not clearly observed, but were expected to be expressed at lower levels given that they are relatively minor components of BMCs both in natural and minimal, recombinant BMCs [12, 24, 25]. In the Eut strains, only EutS and EutL were clearly observed, but as expression levels were not very high, it was difficult to ascertain the presence of the other Eut shell proteins.

Growth curves for the Eut strains under both rhamnose only induction and co-induction were the same, showing significant toxicity (manifested as an arrest or decline in OD_600_) of the EutC-E in both strains and no apparent growth shielding by co-expression of the Eut BMC shell proteins. Western blot analysis for the Ec2985 strain (EutC-E + full Eut), however, revealed a slight, but consistently higher EutC-E expression in triplicate cultures (119 ± 7%) during co-induction relative to rhamnose only induction, suggesting some potential toxicity shielding with BMC co-expression. In the Ec3185 strain (EutC-E + EutS), no significant difference in EutC-E expression was observed between co-induction versus rhamnose only induction, although significant variability was observed among cultures.

The Ec3086 strain (PduD-E + full Pdu) under rhamnose only induction and co-induction grew similarly to the IPTG only condition, indicating no toxicity under these induction conditions. Western blot analysis showed no difference between PduD-E levels under rhamnose only induction versus co-induction. Assuming all E constructs have similar detection via anti-His_6_, PduD-E was found to be expressed at significantly lower levels than EutC-E and PduP-E. Using OD_600_ as an approximation for cell number, blot intensities normalized to the OD_600_ of the culture suggest that the PduD-E level per cell is likely at least ~fourfold and ~8- to 12-fold lower than the EutC-E and PduP-E levels per cell, respectively. This result may partially explain why no toxicity-related growth arrest was observed with the Ec3086 strain, unlike all the other strains. It is also possible that PduD-E is less toxic than EutC-E and PduP-E.

The Ec3087 strain (PduP-E + full Pdu) was the only strain to exhibit improved growth under co-induction compared to rhamnose only induction, indicating some toxicity shielding with co-expression of the full Pdu BMC system. The maximum growth difference was observed at 3 h, where the co-induction culture had ~20% higher OD_600_ than the rhamnose only culture. Western blot analysis revealed that PduP-E expression in the co-induction culture was 149 ± 15% higher than the rhamnose only culture.

We attempted to construct a negative control plasmid containing the gene for non-tagged E under a *pRha* promoter, which could be expressed in combination with the various empty, recombinant BMC shell systems to ensure that there is no toxicity shielding without a BMC targeting tag. However, we found that this plasmid could not be cloned without random point mutations that inactivated the E protein (data not shown). This result suggested that placement of N-terminal BMC targeting tags on E reduced its toxicity enough for stable cloning.

### Optimization of PduP-E expression with Pdu BMCs

The Ec3087 strain appeared to have the highest level of E expression per L of culture and exhibited some toxicity shielding upon co-expression of BMCs. Therefore, this strain was chosen for further study and optimization. First, the effect of IPTG timing and concentration was tested (Additional file 1: Figures S3, S4). Similar growth and PduP-E expression levels were found when IPTG was added at the start of growth (3 h prior to rhamnose induction) versus together with rhamnose, indicating that IPTG can be added to the culture at any time prior to rhamnose induction. Induction with 0.5 mM IPTG exhibited the highest expression of PduP-E, compared to 0.1 and 0.2 mM IPTG. Higher concentrations of IPTG were not tested because levels of BMC shell proteins were already highly over-expressed compared to PduP-E and too high expression of BMC shell proteins has been shown to lead to inclusion body formation [26]. Thus, use of 0.5 mM IPTG at the start of growth was chosen for induction of BMC shell proteins in subsequent experiments.

**Fig. 2:**
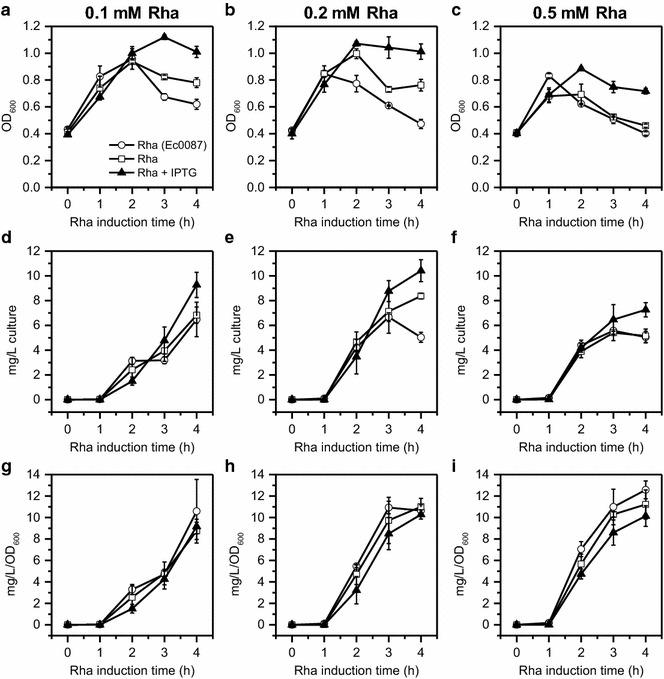
Growth and PduP-E expression in Ec3087 and Ec0087 under different induction conditions. Ec0087 induced with rhamnose only (*open circle*); Ec3087 induced with rhamnose only (*open square*); and Ec3087 co-induced with rhamnose and IPTG (*filled triangle*). IPTG was added at t = −3 h to 0.5 mM. Rhamnose was added at t = 0 to 0.1 mM in **a**, **d**, **g**, to 0.2 mM in **b**, **e**, **h**, and to 0.5 mM in **c**, **f**, **i**. *Error bars* represent data from 3 biological replicates. **a**–**c** Growth of cells. **d**–**f** Amounts of PduP-E expressed per L of culture determined by quantitative Western blot (see Additional file 1: Figure S5 for raw data). **g**–**i** Amounts of PduP-E expressed per L of culture normalized to OD_600_

PduP-E expression levels were then optimized by varying the amount of rhamnose added to the culture (0.1, 0.2, and 0.5 mM) in the presence of 0.5 mM IPTG induction and monitoring PduP-E expression (in mg/L of culture) over time by quantitative Western blot (Fig. 2; Additional file 1: Figure S5). For comparison, the amounts of PduP-E produced in Ec3087 in the absence of IPTG and in control strain Ec0087 harboring only the PduP-E plasmid were also determined under the same conditions. Ec0087 controls for the effect of harboring the full Pdu BMC plasmid on PduP-E expression.

Growth curves (Fig. 2a–c) generally showed that under each rhamnose concentration, the most toxicity occurred with Ec0087, followed by Ec3087 under rhamnose only induction. The least toxicity occurred with Ec3087 under co-induction. The only exception to this trend was observed at 0.5 mM rhamnose, where Ec0087 and Ec3087 under rhamnose only induction exhibited similar degrees of toxicity. Nonetheless, these results demonstrated that toxicity shielding by BMC co-induction occurred across different rhamnose induction concentrations. As expected, faster onset of toxicity occurred with higher rhamnose concentration for corresponding strains and IPTG induction conditions, although differences were minimal between the 0.1 and 0.2 mM rhamnose conditions.

Amounts of PduP-E per L of cell culture over time (Fig. 2d–f) show maximum PduP-E levels (~9–10 mg/L) with Ec3087 under 0.1 and 0.2 mM rhamnose co-induction with IPTG at 4 h post-rhamnose induction. The yield under 0.5 mM rhamnose co-induction with IPTG was ~30% lower. PduP-E levels in Ec3087 under rhamnose only induction were ~20–30% lower than the levels in their respective co-induction condition at 4 h post-induction across rhamnose concentrations, again demonstrating higher production of PduP-E with co-induction of the BMC shell proteins. For the Ec0087 control, the PduP-E levels at 4 h post-induction under 0.1 and 0.5 mM rhamnose were the same as for Ec3087 under rhamnose only induction, but was ~40% lower under 0.2 mM rhamnose. Overall, Ec3087 under 0.2 mM rhamnose co-induction with IPTG had a ~twofold higher PduP-E expression level compared to Ec0087 under 0.2 mM rhamnose.

To examine the kinetics of PduP-E expression over time, the amount of PduP-E in the total culture was normalized to OD_600_, which was used as an approximation for cell number. Colony forming unit (CFU) counts were not used because they do not include dead cells that had synthesized PduP-E, but were no longer viable. OD_600_ was therefore chosen as it more closely reflects all cells, alive or dead, that produced PduP-E. The OD_600_ normalized amounts of PduP-E per L of culture over time (Fig. 2g–i) showed, as expected, that PduP-E accumulated faster in cells induced with higher concentrations of rhamnose for corresponding strains and IPTG induction conditions. This effect was most pronounced at 2 h post-induction, when toxicity observed in the growth curves was limited. In all strains, the maximum PduP-E accumulation appeared to be ~11 mg/L of OD_600_ of 1, which is where the curves appear to level off.

Under respective 0.2 and 0.5 mM rhamnose induction conditions, Ec0087 appeared to accumulate PduP-E at a significantly faster rate than Ec3087 under co-induction. Ec3087 under rhamnose only induction was intermediate, although error bars appear to overlap with both conditions. With 0.1 mM rhamnose, the same trend is observed at 2 h post-induction, but by 3 and 4 h post-induction, the PduP-E accumulation levels were similar. In general, these trends correlate with growth trends and support that faster accumulation of PduP-E results in faster induced toxicity and thus lower overall yield. Ec0087 likely accumulates PduP-E at a faster rate than Ec3087 because the copy number of the PduP-E plasmid in Ec3087 is lower than in Ec0087. The presence of the BMC plasmid in Ec3087 likely lowers the copy number of the PduP-E plasmid because they both have the same origin of replication and compete for copy number.

### Co-expression of full and truncated sets of BMC proteins provide toxicity shielding for PduP-E

**Fig. 3:**
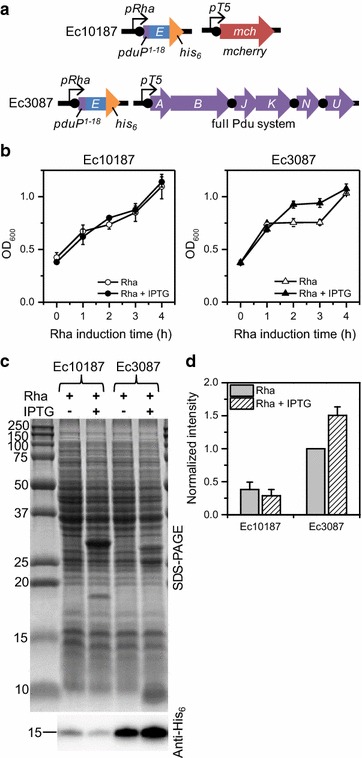
Co-expression of PduP-E with mCherry (Ec10187) versus with the Pdu BMC system (Ec3087). **a** Genetic scheme for the different strains. *Black circles* represent ribosome binding/re-initiation sites. **b** Growth curves for each strain under different induction conditions. *Open shapes* induction with 0.1 mM rhamnose only at t = 0; *closed shapes* co-induction with 0.5 mM IPTG at t = −3 h and 0.1 mM rhamnose at t = 0. *Error bars* represent data from 3 replicates. **c** SDS-PAGE and anti-His_6_ Western blot at 4 h post-rhamnose induction from 1 representative growth. The equivalent of 50 and 2 µL of original culture were analyzed by SDS-PAGE and Western blot, respectively. Sizes of molecular weight standards in kDa are shown on the *left*. **d** Relative Western blot intensities compared to Ec3087, Rha only. *Gray bars* rhamnose only induction; *striped bars* IPTG and rhamnose co-induction. *Error bars* represent data from 3 replicates from the different growths in **b** (see Additional file 1: Figure S6 for raw data)

Next, we sought to confirm that that the toxicity shielding observed in Ec3087 cells during co-induction with rhamnose and IPTG is specifically due to co-expression of Pdu BMC proteins and not due to a reduced rate of PduP-E expression resulting from significant co-expression of any other protein. To this end, we constructed plasmid pMCY101, where *mcherry* is expressed under the T5 promoter instead of *pduABJKNU*, and co-transformed this plasmid with the PduP-E plasmid to produce strain Ec10187. Growth and PduP-E expression in Ec10187 and Ec3087 under 0.1 mM rhamnose only induction and co-induction with IPTG was examined (Fig. 3; Additional file 1: Figure S6). Cells were grown on the same day to minimize variation in growth conditions.

Growth curves for Ec10187 revealed no growth difference between co-induction and rhamnose only induction and demonstrated a similar degree of toxicity as the growth curve for Ec3087 under rhamnose only induction (Fig. 3b; Additional file 1: Figure S7). In contrast, Ec3087 under co-induction showed improved growth at 2 and 3 h post-induction. Anti-His_6_ Western blot analysis of equivalent volumes of culture at 4 h post-induction (Fig. 3c, d) also showed no significant difference in PduP-E expression for Ec10187 under rhamnose only induction versus co-induction; PduP-E levels were 38 ± 11% and 29 ± 10%, respectively, of that of Ec3087 under rhamnose only. In contrast, PduP-E levels in Ec3087 under co-induction were 50 ± 13% higher than under rhamnose only induction. These results indicate that toxicity shielding in growth and higher overall PduP-E yield is associated with co-expression of BMC shell proteins. We note that PduP-E levels were found to be significantly lower in Ec10187, suggesting that copy number of the PduP-E plasmid is even lower in this strain compared to Ec3087. However, lower levels of PduP-E in Ec10187 likely does not affect the major conclusion that co-expression of PduP-E with mCherry does not improve overall expression of PduP-E.

**Fig. 4:**
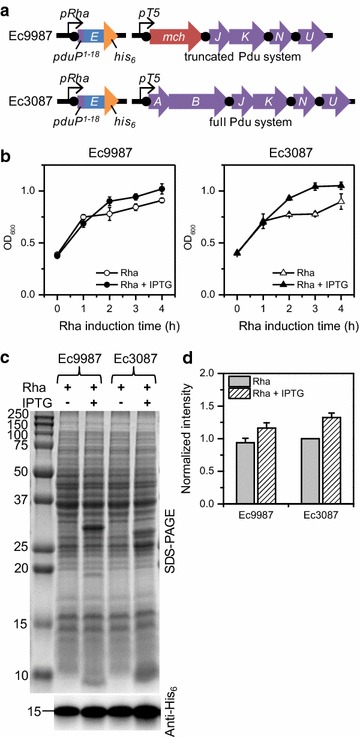
Co-expression of PduP-E with a truncated (Ec9987) versus full (Ec3087) Pdu BMC system. **a** Genetic scheme for the different strains. *Black circles* represent ribosome binding/re-initiation sites. **b** Growth curves for each cell strain under different induction conditions. *Open shapes* induction with 0.1 mM rhamnose only at t = 0; *closed shapes* co-induction with 0.5 mM IPTG at t = −3 h and 0.1 mM rhamnose at t = 0. *Error bars* represent data from 3 replicates. **c** SDS-PAGE and anti-His_6_ Western blot at 4 h post-rhamnose induction from 1 representative growth. The equivalent of 50 and 2 µL of original culture were analyzed by SDS-PAGE and Western blot, respectively. Sizes of molecular weight standards in kDa are shown on the *left*. **d** Relative Western blot intensities compared to Ec3087, Rha only. *Gray bars* rhamnose only induction; *striped bars* IPTG and rhamnose co-induction. *Error bars* represent data from 3 replicates from the different growths in **b** (see Additional file 1: Figure S8 for raw data)

Finally, we examined the effect of co-expressing a truncated Pdu BMC shell system (deficient in forming intact BMCs [8]) on expression of PduP-E by constructing pMCY99, which expresses *mcherry* with *pduJKNU* from the T5 promoter. Ec9987 harboring pMCY99 and the PduP-E plasmid was again compared to Ec3087 during 0.1 mM rhamnose only induction versus co-induction with IPTG (Fig. 4; Additional file 1: Figure S8), where cells were grown on the same day to minimize growth variability. Ec9987 behaved very similarly to Ec3087, but the effects on growth and PduP-E expression were slightly muted. At 3 h post-induction, where growth shielding was most apparent, Ec3087 under co-induction had a ~34% increase in OD_600_ over rhamnose only induction, but Ec9987 had only a ~12% increase (Fig. 4b). At 4 h post-induction, Western blots showed that Ec3087 had a 33 ± 7% increase in PduP-E levels under co-induction compared to rhamnose only induction (Fig. 4c, d). For Ec9987, the increase was 24 ± 13%, which was within error of the increase in Ec3087. PduP-E levels in both strains under rhamnose only induction were within error of each other. These results suggest that expression of *pduJKNU* alone can shield toxicity of PduP-E and the formation of BMC-like particles (requiring the presence of PduAB [8]) is not necessary.

### PduP-E co-purifies with isolated BMCs

We next examined whether PduP-E is associated with recombinant BMCs in vivo. BMCs were isolated from Ec3087 cells co-induced with 0.5 mM IPTG and 0.1, 0.2, or 0.5 mM rhamnose for 3 h according to previous methods [20]. As a positive control, BMCs were also isolated from Ec3090 cells harboring both pMCY90 (which expresses a N-terminal PduP-tagged and C-terminal His_6_-tagged mCherry, PduP-mCherry) and pMCY30 (which expresses *pduABJKNU*). To determine the amount of contaminating PduP-E (PduP-E that is purified from the BMC isolation, which is not BMC-associated), a mock isolation was also performed using Ec0087 cells. Ec3090 and Ec0087 cells were induced with 0.2 mM rhamnose and 0.5 mM IPTG where appropriate.

**Fig. 5:**
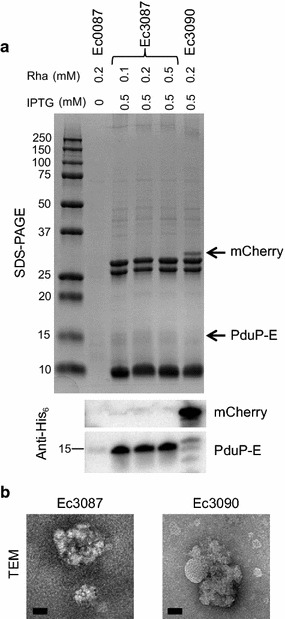
Co-isolation of PduP-tagged protein with purified BMCs. **a** SDS-PAGE and anti-His_6_ Western blot of purified BMCs from Ec3087. As negative and positive controls, BMCs were also purified from Ec0087 (no BMCs) and Ec3090 (PduP-mCherry + full Pdu), respectively. BMCs were purified from cells at 3 h post-rhamnose induction. Induction conditions are identified at the top of the gel. 10 µg of BMCs from Ec3087 and Ec3090 and 1 µg of mock BMCs from Ec0087 were loaded on the SDS-PAGE. 1 µg of each BMC sample was analyzed by Western blot. Sizes of molecular weight standards in kDa are shown on the left. **b** TEM analysis of isolated BMCs from Ec3087 and Ec3090. *Scale bars* are 50 nm

Isolated BMCs were analyzed by SDS-PAGE, anti-His_6_ Western blot, and TEM (Fig. 5). As expected, isolated BMCs across samples primarily contain shell proteins corresponding to sizes for PduB, PduB′, PduA, and PduJ by SDS-PAGE. A light band at ~20 kDa can also be observed, which corresponds to the M_w_ for PduK. TEM analysis revealed that isolated BMCs from Ec3087 and Ec3090 cells were morphologically similar with irregular shapes of up to ~200 nm in diameter. While these recombinant BMCs are irregularly shaped compared to native BMCs, they are similar to other isolated recombinant BMCs observed in prior studies [12, 27]. SDS-PAGE and Western blot analysis, furthermore, revealed that PduP-mCherry (~31 kDa) was co-isolated with the BMC sample from Ec3090, and that PduP-E was co-isolated with all three BMC samples from Ec3087, although PduP-E was not clearly observed by SDS-PAGE. Very little contaminating PduP-E was found in the mock BMC isolation from Ec0087, <0.8% of that isolated from Ec3087 based on quantitative Western blot analysis (Fig. 5a; Table 2; Additional file 1: Figure S9). Interestingly, the amounts of PduP-E co-isolated per mg of total BMC protein were very similar in all three BMC samples from Ec3087, despite the differences in rhamnose induction concentrations and total BMC yields: 12–14 µg of PduP-E per mg of total BMC protein (Table 2). This result suggested that PduP-E was fully loaded into the isolated BMCs and increasing rhamnose concentrations, corresponding to increasing expression of PduP-E, did not improve load.

**Table 2:** Amounts of PduP-tagged protein isolated with BMCs

Strain	Induction conditions	Total PduP-tagged protein in µg (% in total lysate)^a^			Total BMC yield (mg)^b^	PduP-tagged protein/BMCs (µg/mg)
		Lysate	Soluble fraction	Isolated BMCs		
Ec0087	0.2 mM Rha	1300 (100%)	75 (5.8%)	0.063 (0.0049%)	0.062	1.0
Ec3087	0.1 mM Rha + 0.5 mM IPTG	1300 (100%)	380 (28%)	18 (1.4%)	1.4	14
Ec3087	0.2 mM Rha + 0.5 mM IPTG	1700 (100%)	170 (10%)	8.7 (0.52%)	0.74	12
Ec3087	0.5 mM Rha + 0.5 mM IPTG	1100 (100%)	120 (11%)	12 (1.1%)	0.94	13
Ec3090	0.2 mM Rha + 0.5 mM IPTG	29,000 (100%)^c^	22,000 (75%)^c^	73 (0.25%)^c^	0.88	39^a^

^a^Amounts of PduP-tagged proteins were determined by quantitative Western blots using anti-His_6_ (see Additional file 1: Figure S9 for raw data)

^b^Total protein content was determined by Bradford assay using BSA as a standard

^c^mg of PduP-mCherry was estimated based on a PduP-E standard curve, assuming anti-His_6_ detects an equal number of PduP-E and PduP-mCherry molecules [i.e., 1 ng of PduP-E (14.8 kDa) detected is equivalent to 2.1 ng of PduP-mCherry (31.2 kDa) detected]

Of the total expressed PduP-E in the cell lysate, only 10–28% of the PduP-E was found in the soluble fraction and only ~0.5–1.4% was found to be co-isolated with BMC samples from Ec3087 (Table 2). Given that PduP-E is a membrane protein, this result shows that most protein was still inserted into the membrane and not sequestered into the soluble fraction by BMC proteins. However, in the negative control Ec0087 cells, a lower percentage (5.8%) of the PduP-E was found in the soluble fraction, suggesting that the presence of BMC shell proteins increased the amount of PduP-E found in the soluble fraction. In the positive control, the amount of PduP-mCherry co-isolated with BMCs was similarly low at 0.25% of the total PduP-mCherry in the cell lysate. Here, however, the expression of PduP-mCherry was observed to be much higher than expression of the BMC shell proteins, indicating an excess of PduP-mCherry (Additional file 1: Figure S10). Thus, it was not surprising that the percentage of PduP-mCherry co-isolated with BMCs was low. Given the size heterogeneity and irregular shapes of the isolated BMCs observed by TEM, there is likely poor loading of PduP-tagged protein into BMCs due to poor formation of intact BMCs in recombinant systems. SDS-PAGE analysis after each centrifugation step in the BMC isolation also revealed loss of BMC shell proteins throughout the purification (Additional file 1: Figure S10), suggesting that a large proportion of BMC proteins do not properly form intact BMCs, although we cannot rule out that the isolation procedure was also inefficient. This result at least partially explains the variability in the total yields of BMCs isolated from cells, which ranged from 0.88 to 1.4 mg of BMC protein per 200 mL of culture.

### PduP-E can be purified from whole cells and isolated BMCs

**Fig. 6:**
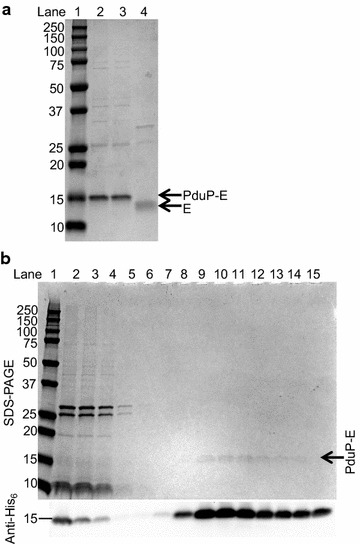
Purified PduP-E and non-tagged E from whole cells and purified BMCs. **a** SDS-PAGE analysis of PduP-E purified from whole cells and non-tagged E after Factor Xa proteolysis. *Lane 1* molecular weight markers with sizes of standards in kDa on the left. *Lane 2* PduP-E purified from Ec3087 induced with 0.1 mM rhamnose only. *Lane 3* PduP-E purified from Ec3087 co-induced with 0.1 mM rhamnose and 0.5 mM IPTG. *Lane 4* non-tagged E after Factor Xa proteolysis. *Lanes 2*–*4* were loaded with 1 µg of protein. **b** SDS-PAGE and anti-His_6_ Western blot analysis during purification of PduP-E from purified BMCs. *Lane 1* molecular weight markers. *Lane 2* purified BMCs from Ec3087 co-induced with rhamnose and IPTG (starting material). *Lane 3* flow through after binding to the Ni–NTA column. *Lanes 4*–*6* sequential wash fractions containing 20 mM imidazole. *Lanes 7*–*15* sequential elution fractions containing 200 mM imidazole

PduP-E was purified from Ec3087 cells under 0.1 mM rhamnose only induction or co-induction with IPTG to ~80% purity (Fig. 6a). Total yield of PduP-E from the co-induced culture was 270 µg/L, which was 33% higher than the yield from the rhamnose only culture, 200 µg/L (Table 3). These yields reflect the expression levels of PduP-E in the cells, ~9 and ~7 mg/L, respectively for co-induction and rhamnose only induction (Fig. 2), however, recovery from the cells was clearly low. One primary factor for low recovery was that only the most concentrated (10 µM) and purest fractions were collected from the Ni–NTA column. Additional fractions were not collected because PduP-E could not be concentrated without precipitation (data not shown). Elution of PduP-E was also inefficient, trailing off from the column in several fractions (data not shown). Additionally, extraction of E by the EBB detergent was shown to be only ~50% from *E. coli* membranes [16], which also may have attributed to the poor recovery.

**Table 3:** Purified PduP-E yields and activity

Sample	Induction conditions	Total yield (µg/L of culture)	Activity (% growth inhibition after 6 h)^a^
PduP-E from Ec3087	0.1 mM Rha	200	72 ± 3
PduP-E from Ec3087	0.1 mM Rha + 0.5 mM IPTG	270	70 ± 5
EBB buffer control	N/A	N/A	34 ± 6
E after Factor Xa cleavage	0.1 mM Rha + 0.5 mM IPTG	200	49 ± 6
Factor Xa control	N/A	N/A	28 ± 2

^a^Activity is presented as the percent that *B. licheniformis* growth is inhibited by 0.5 µM of E in culture compared to normal growth after 6 h based on OD_600_

Following purification of PduP-E, the PduP-tag could be removed from E via Factor Xa proteolysis in a highly efficient fashion. After cleavage, 0.72 µg of non-tagged E was recovered per µg of PduP-E digested (0.88 µmol non-tagged E recovered per µmol PduP-E digested, 88% yield). The non-tagged E was found to no longer bind to Ni–NTA resin, allowing for partial removal of the major contaminant at ~25 kDa by incubation with Ni–NTA resin. The ~25 kDa contaminant is likely SlyD, a peptidyl-prolyl *cis*–*trans* isomerase, that has been shown to interact with E and is required for lysis by E in vivo [22, 28]. SlyD has a histidine rich C-terminal region and therefore is commonly purified as a contaminant with Ni–NTA purifications [29]. The final yield of non-tagged E from Ec3087 cells under co-induction was 200 µg/L (~80% pure), which is a ~sevenfold improvement to a previously reported yield of 27 µg/L (84% pure) [16]. We note, however, that we extracted PduP-E from total lysate including the soluble fraction where BMCs would be localized to maximize yield, whereas Young and coworkers extracted E from only the membrane fraction [16]. Thus, our yields may be inherently higher based on our modified method. We demonstrated that PduP-E could be purified from isolated BMCs by extraction with EBB followed by Ni–NTA purification (Fig. 6b). About 50% of the PduP-E associated with isolated BMCs bound to the column after EBB extraction based on Western blot intensity.

Finally, the purified PduP-E and non-tagged E were tested for cell growth inhibition activity against *B. licheniformis* [22] (Table 3; Additional file 1: Figure S11). While the control containing EBB buffer in culture inhibited *B. licheniformis* growth by 34% after 6 h growth compared to normal growth, 0.5 µM PduP-E from both cell sources inhibited growth by ~70%. For the non-tagged E, the control containing PMSF-inactivated Factor Xa in EBB buffer inhibit growth by 28%, while 0.5 µM non-tagged E inhibited growth to a higher degree, by 49%. Here, the Factor Xa control grew better than the EBB only control, for reasons that are unclear. However, this result may explain why non-tagged E inhibited growth to a lesser degree than PduP-E. Overall, purified PduP-E and non-tagged E were observed to be active in inhibiting *B. licheniformis* growth, although higher toxicity with non-tagged E was not observed.

## Discussion

In this study, we attempted to encapsulate the ϕX174 toxic lysis protein E in recombinant BMCs to shield its toxicity and enhance its heterologous expression in *E. coli*. Ultimately, we showed that a PduP-tagged E could be expressed at moderately high levels (~6–8 mg/L) and that co-expression with the Pdu BMC shell proteins increased PduP-E levels by 20–50% (up to ~10 mg/L). Purification of PduP-E followed by removal of the PduP-tag by Factor Xa proteolysis led to a final yield of 200 µg of E per L of culture, an nearly order of magnitude (~sevenfold) improvement compared to previously reported yields [16]. This work demonstrates improved expression of a toxic protein through several mechanisms described below, which can be potentially applied to other toxic proteins.

One of the primary modes of improvement in expression was the addition of N-terminal BMC targeting tags onto E, which appeared to partially inactivate E. Two lines of evidence support partially inactivated E. First, we were not able to clone non-tagged E under the *pRha* promoter after many attempts, while the N-terminally tagged constructs were easily cloned (data not shown). Here, leaky expression of non-tagged E from the *pRha* promoter, which utilizes the native *E. coli* polymerase for expression (unlike T7 systems) [30], likely resulted in significant cell death and the inability to obtain positive clones. Second, growths expressing N-terminally tagged E constructs did not immediately result in toxicity observed in the growth curves, despite significant expression levels of the tagged E constructs (Fig. 1). In the case of Ec0087 expressing only PduP-E, toxicity was not observed until >1 h after induction, when as much as ~3000 molecules of PduP-E accumulated per cell based on CFU counts (data not shown) and Western blots (Fig. 2c, f, i). This result contrasts with previously reported expression of non-tagged E, in which toxicity was observed within 10–20 min when ~500 molecules of E accumulated per cell [16, 31]. The N-terminal region of E has been shown to be responsible for toxicity of E in *E. coli* [32]; it contains the only transmembrane domain in E with key residues found to be important in binding to MraY [22, 33]. Thus, the N-terminal BMC targeting tags may perturb the proper orientation of E in the membrane and/or inhibition of MraY and thus mitigate E toxicity. Additionally, protein fusions containing BMC targeting tags have been shown to self-aggregate even in the absence of BMC shell proteins [34], which may also be detrimental to E activity.

We, however, did not find non-tagged E to be more toxic than PduP-E in cell inhibition assays. This result may have been due to use of *B. licheniformis* in the assay, whose MraY may be relatively insensitive to E based on 92% sequence identity to insensitive MraY in *B. subtilis* [35]. Given the importance of the N-terminal region of E, the PduP-E construct was carefully designed so that removal of the PduP-tag via Factor Xa protease (which cleaves following the R residue in its recognition sequence IEGR [36]) resulted in the native N-terminal E sequence. The C-terminal His_6_-tag was not removed as previous studies showed that C-terminal His_6_ tags did not interfere with the activity of expressed E in killing *E. coli* [16, 31]. Thus, we would have expected our non-tagged E construct to be active against *E. coli*. However, *E. coli* cell inhibition assays were unsuccessful (data not shown), presumably due to the inability of exogenously added PduP-E/non-tagged E to traverse the outer membrane to access MraY in the inner membrane.

Another significant improvement in PduP-E expression was observed with the double plasmid Ec3087 strain compared to the single plasmid Ec0087 strain, even without co-induction with IPTG. Here, the presence of the BMC plasmid likely reduced the copy number of the PduP-E plasmid, allowing for slower and thus better overall expression of PduP-E (Fig. 2), which has been observed in other systems when lower copy number plasmids are used [1]. Given that the two plasmids have the same origin of replication, this finding is not unexpected. We used the same *pUC* origin of replication, which provides ~500–700 copies per cell [1], to maximize the levels of both BMC and N-terminally tagged E protein. We recognize that consistency in plasmid copy number may be a limitation of this study, which was observed with the different levels of tagged E protein among the different double plasmid strains (Ec2985, Ec3185, Ec3086, Ec3087, and Ec10187) upon rhamnose only induction, despite use of the same *pRha* promoter. To limit day-to-day growth and expression variability in copy number, we conducted comparison studies in this paper on the same day and determined E construct amounts on the same Western blot to make direct comparisons between strains.

Finally, significant 20–50% improvements in growth and PduP-E expression levels were observed with co-expression of Pdu BMC shell proteins. Similar improvements were also observed with a truncated BMC system expressing *mcherry* + *pduJKNU* (Fig. 4). This result suggests that all components for a fully formed BMC are not required for enhanced PduP-E expression. Truncated BMC systems have been shown to form lattice-like and filamentous structures in the cytoplasm of *E. coli* [8], and thus targeting to these structures may have aided in sequestering PduP-E away from MraY in the membrane. Given that PduP-tag has been shown to interact with the α-helical C-termini of PduA and PduJ in *S. enterica* and PduK in *C. freundii* [6, 7], only BMC proteins that interact with PduP-tag appear to be necessary for the enhancement effect. Experiments with the Ec10187 strain co-expressing mCherry instead of the BMC proteins demonstrate that the enhancement in PduP-E expression only occurs with BMC shell proteins (Fig. 3). Furthermore, lack of significant enhancement in other BMC systems (Fig. 1) suggests a specificity with PduP-tag, which will require further study.

Despite the overall improvement in PduP-E production in culture, we observed that the same maximum PduP-E accumulation (~11 mg/L of OD_600_ = 1 culture) was reached in both Ec0087 and Ec3087, indicating that complete encapsulation of PduP-E is minimal and any cellular protection by sequestration of PduP-E is eventually overcome to induce toxicity. Lack of complete encapsulation was supported by our finding that only ~1% of the total expressed PduP-E was co-isolated with purified BMCs. Poor encapsulation was most likely due to poor formation of intact BMCs, which was supported by TEM data showing irregular shapes and sizes of the isolated BMCs. Poor formation of Eut BMCs relative to Pdu BMCs may also explain why significantly enhanced EutC-E expression in the Eut BMC system was not observed. Recently, EutQ was implicated in helping to form multiple Eut BMCs in *E. coli* and thus may be required for better Eut BMC formation [37]. Another reason that relatively few PduP-E molecules were encapsulated may be that insertion of PduP-E into the membrane and its subsequent inhibition of MraY is fast relative to encapsulation. Given that many different shell proteins need to come together to form a BMC, encapsulation is likely to be slow compared to MraY binding. Molecular stoichiometry based on 2D gel electrophoresis analysis of natural Pdu BMCs isolated from *S. enterica* suggests that 19.5, 11.5, 12.5, 30.5, 2.5, and 3 molecules of PduA, B, B’, J, K, and U, respectively, are needed per 4 molecules of PduP for complete BMC formation [24]. Clearly, more efficient methods of encapsulation with better formation of recombinant BMCs need to be developed.

Other studies have also shown that protein encapsulation can shield toxicity of interior proteins. Hilvert and coworkers demonstrated that HIV protease could be encapsulated in compartments formed by an engineered lumazine synthase from *Aquifex aeolicus* containing negatively charged residues on the luminal side of its formed cages [4]. Encapsulation was achieved using an evolved, positively-charged C-terminal targeting tag on HIV protease. Similar strategies can also be utilized for other designated encapsulin nanocompartments, such as linocin-like encapsulin from *Thermotoga maritima*, using C-terminal targeting sequences [38]. We attempted to use the encapsulin system from *T. maritima* to encapsulate E. However, we were not able to clone an E fusion with the required C-terminal targeting tag, presumably because of toxicity of the exposed, non-tagged N-terminal region of E (data not shown) [22, 32]. Thus, only N-terminally tagged E constructs and associated encapsulation systems appear useful in mitigating the toxicity of E, demonstrating the need to test different types of encapsulating systems for specific target proteins.

Overall, our results show that co-expression of BMC shell proteins with PduP-E increased PduP-E expression levels, although expression was also improved by addition of N-terminal tags to E. The methods described herein have the potential to enhance expression of other toxic proteins, by sequestration of the protein away from its target of toxicity. This work showcases yet another application of re-directed BMC systems, which have also recently been used to engineer strains to produce higher amounts of ethanol and 1,2-propanediol [7, 34], and thus demonstrates the potential of re-directed BMCs to enhance engineered microbes for defined functions.

## Conclusions

Here, we report the enhanced expression of toxic lysis protein E from bacteriophage ϕX174 in *E. coli* via a re-directed BMC targeting system. E was tagged with various N-terminal BMC targeting sequences, which not only targeted the protein for association with BMC shell proteins, but also reduced its toxicity to allow for its expression and accumulation. A PduP-tagged E system appeared to be the best system with the highest expression of E protein, which was further enhanced by 20–50% by co-expression of Pdu BMC proteins. PduP-E could be optimally expressed at ~10 mg/L and purified by Ni–NTA affinity chromatography to 270 µg/L. Non-tagged E was obtained by Factor Xa proteolysis for a final yield of 200 µg of E per L. Overall, the results demonstrate the utility of re-directed BMC systems to enhance toxic protein yields in recombinant *E. coli* systems.

## Additional file

**Additional file 1: Table S1.** Primers used in this study. **Figure S1.** Plasmid maps for pMCY29 and pMCY30. **Table S2.** Codon-optimized sequences for pEutC, pPduD, and pPduP. **Figure S2. **Raw anti-His_6_ Western blot data represented in Fig. 1d. **Figure S3.** Effect of timing of IPTG induction (BMC expression) on expression of PduP-E. **Figure S4. **Effect of different IPTG concentrations on expression of PduP-E. **Figure S5.** Raw anti-His_6_ Western blot data represented in Fig. 2. **Figure S6. **Raw anti-His_6_ Western blot data represented in Fig. 3d. **Figure S7. **Growth curves for Ec10187 and Ec3087 under IPTG only and rhamnose only induction. **Figure S8. **Raw anti-His_6_ Western blot data represented in Fig. 4d. **Figure S9.** Raw anti-His_6_ Western blot data for amounts presented in Table 2. **Figure S10.** SDS-PAGE analysis of samples during isolation of BMCs. **Figure S11.** *B. lincheniformis* growth inhibition assays for PduP-E and non-tagged E.

## Abbreviations

E: lysis protein E
BMC: bacterial microcompartment
Pdu: 1,2-propanediol utilization
Eut: ethanolamine utilization
OD_600_: optical density at 600 nm
PCR: polymerase chain reaction
GFP: green fluorescent protein
Rha: rhamnose
IPTG: isopropyl-β-d-1-thiogalactopyranoside
TEM: transmission electron microscopy
