# Supplementary material for: Re-directing bacterial microcompartment systems to enhance recombinant expression of lysis protein E from bacteriophage ϕX174 in Escherichia coli
Source: Microb Cell Fact. 2017 Apr 26;16:71. doi: 10.1186/s12934-017-0685-x (PMC5405515; doi:10.1186/s12934-017-0685-x)
Supplement: Supplementary file 1 — Additional file 1: Table S1. Primers used in this study. Figure S1. Plasmid maps for pMCY29 and pMCY30. Table S2. Codon-optimized sequences for pEutC, pPduD, and pPduP. Figure S2. Raw anti-His6 Western blot data represented in Fig. 1d. Figure S3. Effect of timing of IPTG induction (BMC expression) on expression of PduP-E. Figure S4. Effect of different IPTG concentrations on expression of PduP-E. Figure S5. Raw anti-His6 Western blot data represented in Fig. 2. Figure S6. Raw anti-His6 Western blot data represented in Fig. 3d. Figure S7. Growth curves for Ec10187 and Ec3087 under IPTG only and rhamnose only induction. Figure S8. Raw anti-His6 Western blot data represented in Fig. 4d. Figure S9. Raw anti-His6 Western blot data for amounts presented in Table 2. Figure S10. SDS-PAGE analysis of samples during isolation of BMCs. Figure S11. B. lincheniformis growth inhibition assays for PduP-E and non-tagged E. [file 12934_2017_685_MOESM1_ESM.docx]

**Supplementary Material for:**

**Title:** Re-directing bacterial microcompartment systems to enhance recombinant expression of lysis protein E from bacteriophage ϕX174 in *Escherichia coli*.

**Authors:** Mimi C. Yung,^1,^* Feliza A. Bourguet,^1^ Timothy S. Carpenter,^1^ and Matthew A. Coleman^1^

^1^Biosciences and Biotechnology Division, Physical and Life Sciences Directorate, Lawrence Livermore National Laboratory, Livermore, California.

Email addresses for all authors: [yung6@llnl.gov](mailto:yung6@llnl.gov), [bourguet1@llnl.gov](mailto:bourguet1@llnl.gov), [carpenter36@llnl.gov](mailto:carpenter36@llnl.gov), [coleman16@llnl.gov](mailto:coleman16@llnl.gov)

*Corresponding author. Address: Lawrence Livermore National Laboratory, 7000 East Avenue, L-452, Livermore, CA 94550; Phone: (925) 422-7750; Fax: (925) 422-2282; E-mail: [yung6@llnl.gov](mailto:yung6@llnl.gov).

**Table S1. Primers used in this study.**

| Primer name | Sequence (5’ to 3’) |
| --- | --- |
| *lysE_for* | TGAAGGTCGTAGATCTATGGTACGCTGGACTTTGTGG |
| *lysE_rev* | GCGGAACCAGAGATCTCTCCTTCCGCACGTAATTTTTGAC |
| *eutCdBglII* | AGTCCAGCGTACCATACGACCCTCGATCTGACCC |
| *pduDdBglII* | AGTCCAGCGTACCATACGACCCTCGATTTTCATGTCG |
| *pduPdBglII* | AGTCCAGCGTACCATACGACCCTCGATCAGCTGT |
| *dBglII_for* | ATGGTACGCTGGACTTTGTGGGATACCCTCGCTTTC |
| *mcherry_for* | ATGGTGAGCAAGGGCGAG |
| *mcherry_rev* | CTTGTACAGCTCGTCCATGCC |
| *pPduPlysE_for* | GCCCTTGCTCACCATAGATCTCTCCTTCCGCACGT |
| *pPduPlysE_rev* | GACGAGCTGTACAAGCTCGAGCTGGTTCCGCGTGGCTC |
| *mcherry_for2* | CAAGAAGCACTAGGAATGGTGAGCAAGGGCGAG |
| *mcherry_rev2* | TCAGATGTAGGACGGCTTGTACAGCTCGTCCATGC |
| *mcherry_rev3* | TCAACCGCTAGCTTACTTGTACAGCTCGTCCATGCC |
| *pduAup_rev* | TCCTAGTGCTTCTTGTTGCAT |
| *pduBdown_for* | CCGTCCTACATCTGACTAGG |
| *pduUdown_for* | TAAGCTAGCGGTTGACCC |


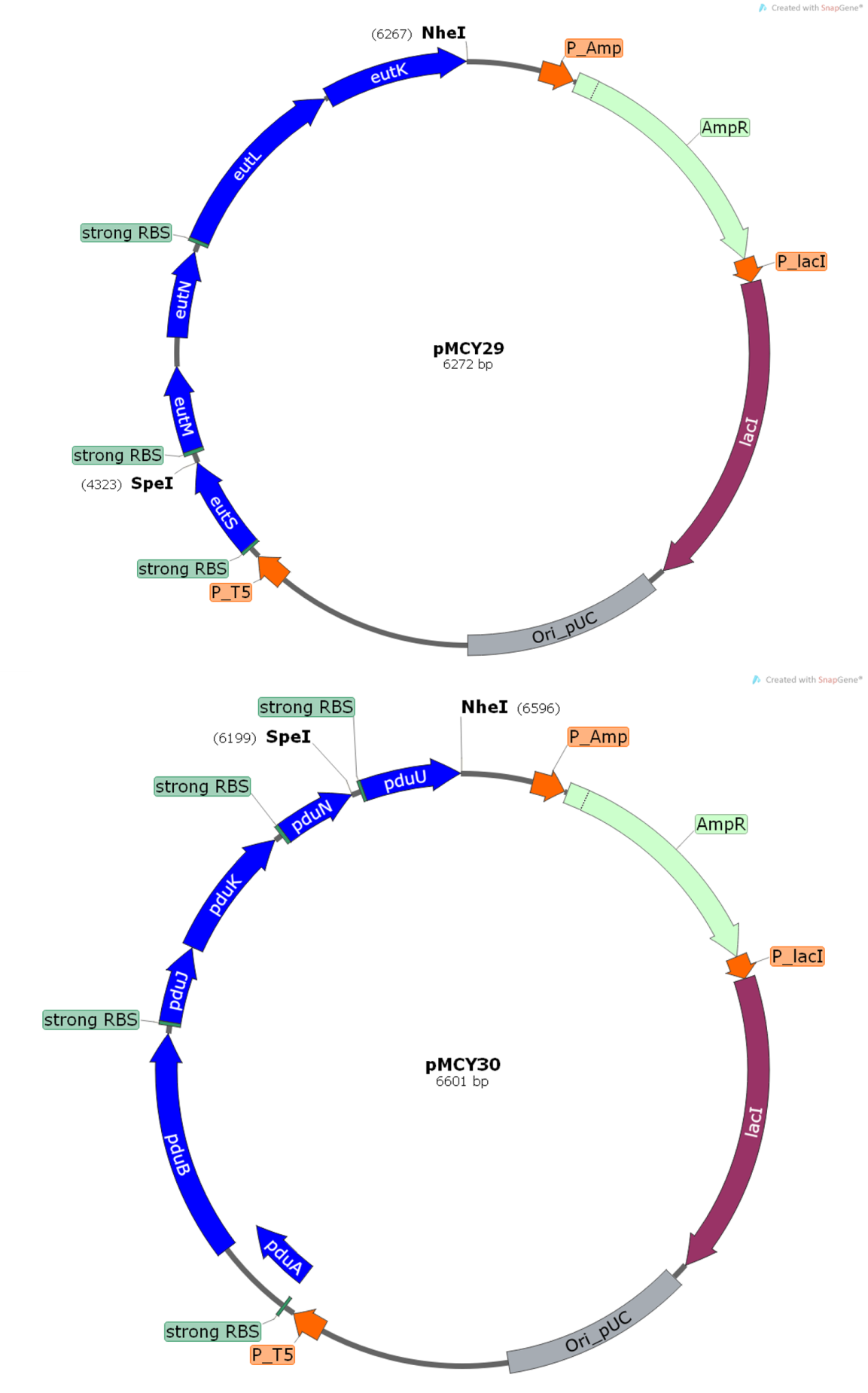


**Figure S1. Plasmid maps for pMCY29 and pMCY30.**

**Table S2. Codon-optimized sequences for pEutC, pPduD, and pPduP.**

| Plasmid | Codon-optimized nucleotide sequence |
| --- | --- |
| pEutC | ATGGACCAGAAACAAATTGAAGAAATTGTGCGTAGCGTTATGGCGTCCATGGGTCAG**ATCGAGGGTCGT**AGATCT*CTGGTCCCGCGTGGCAGC***CACCACCATCATCACCAC** |
| pPduD | ATGGAAATCAACGAAAAGCTGCTGCGTCAGATTATTGAAGATGTGTTGCGCGACATGAAA**ATCGAGGGTCGT**AGATCT*CTGGTTCCGCGTGGCAGC***CATCACCACCACCATCAC** |
| pPduP | ATGAACACGAGCGAGCTGGAAACCCTGATCCGTACCATTTTGAGCGAACAGCTG**ATCGAGGGTCGT**AGATCT*CTGGTTCCGCGTGGCTCC***CACCATCACCACCATCAC** |

Regular font = BMC targeting tag

**Bold** = Factor Xa site

Underlined = *BglII* site for insertion of protein of interest

*Italics* = Thrombin site

**Bold and underlined** = His_6_ tag


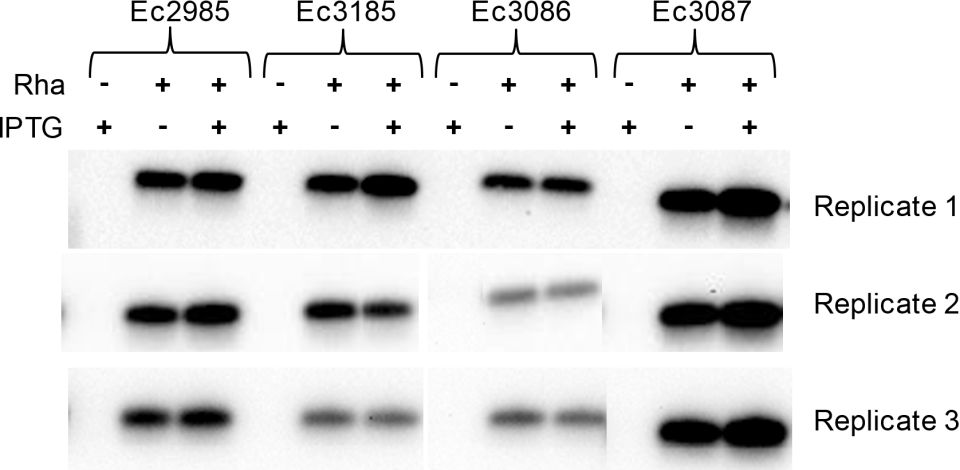


**Figure S2. Raw anti-His_6_ Western blot data represented in Figure 1D.** Each replicate set was performed on the same Western blot. Intensities were determined and normalized to Ec2985, Rha only. Average relative intensities are presented in Figure 1D.


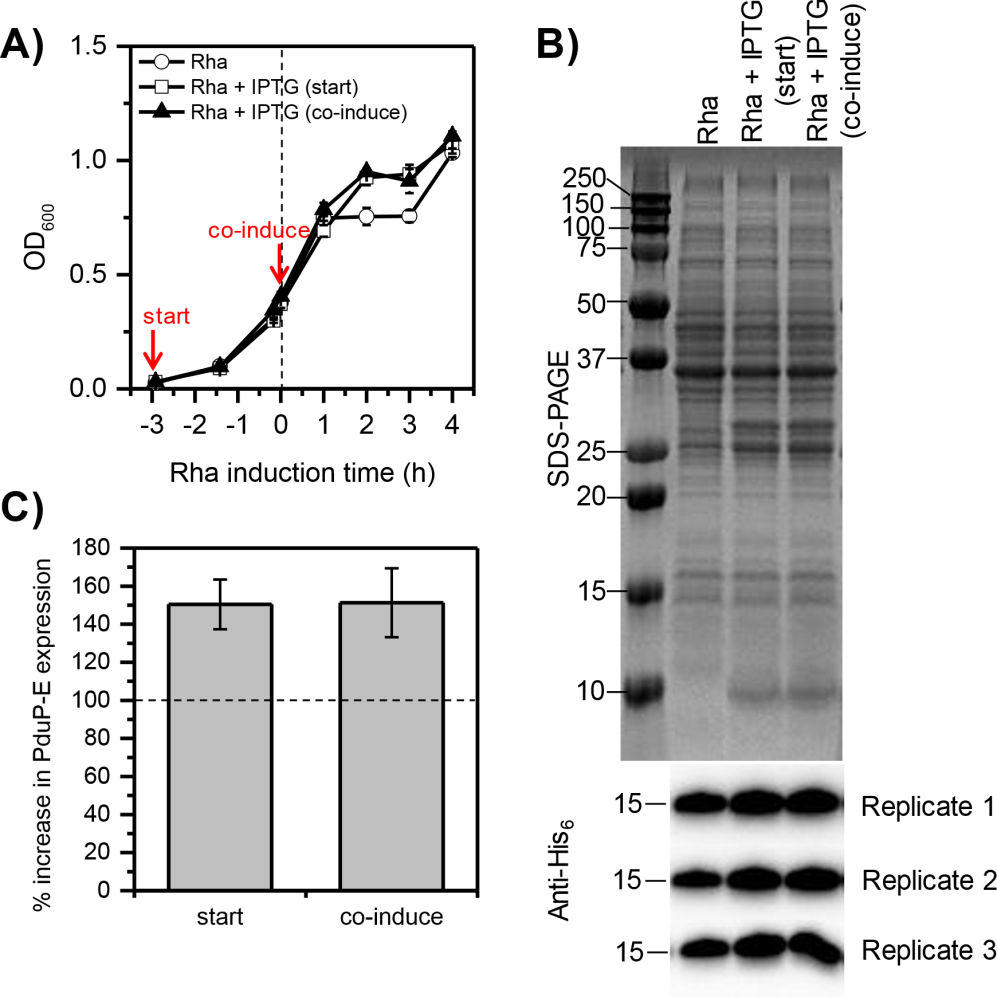


**Figure S3. Effect of timing of IPTG induction (BMC expression) on expression of PduP-E.** (A) Growth curves for Ec3087 under different inductions conditions. Induction with rhamnose only at t=0 (Rha, ○); induction with IPTG at t=-3 h and rhamnose at t=0 (Rha + IPTG (start), □); co-induction with IPTG and rhamnose at t=0 (Rha + IPTG (co-induce), ▲). Error bars represent data from 3 replicates. (B) SDS-PAGE and anti-His_6_ Western blot at 4 h post-rhamnose induction. The equivalent of 50 µL and 2 µL of original culture were analyzed by SDS-PAGE and Western blot, respectively. Sizes of molecular weight standards in kDa are shown on the left. SDS-PAGE analysis from 1 representative growth and Western blot analysis from 3 replicate growths are shown. (C) Relative increase in PduP-E expression compared to the rhamnose only (Rha) condition based on Western blot intensities. Error bars represent data from the 3 replicates.


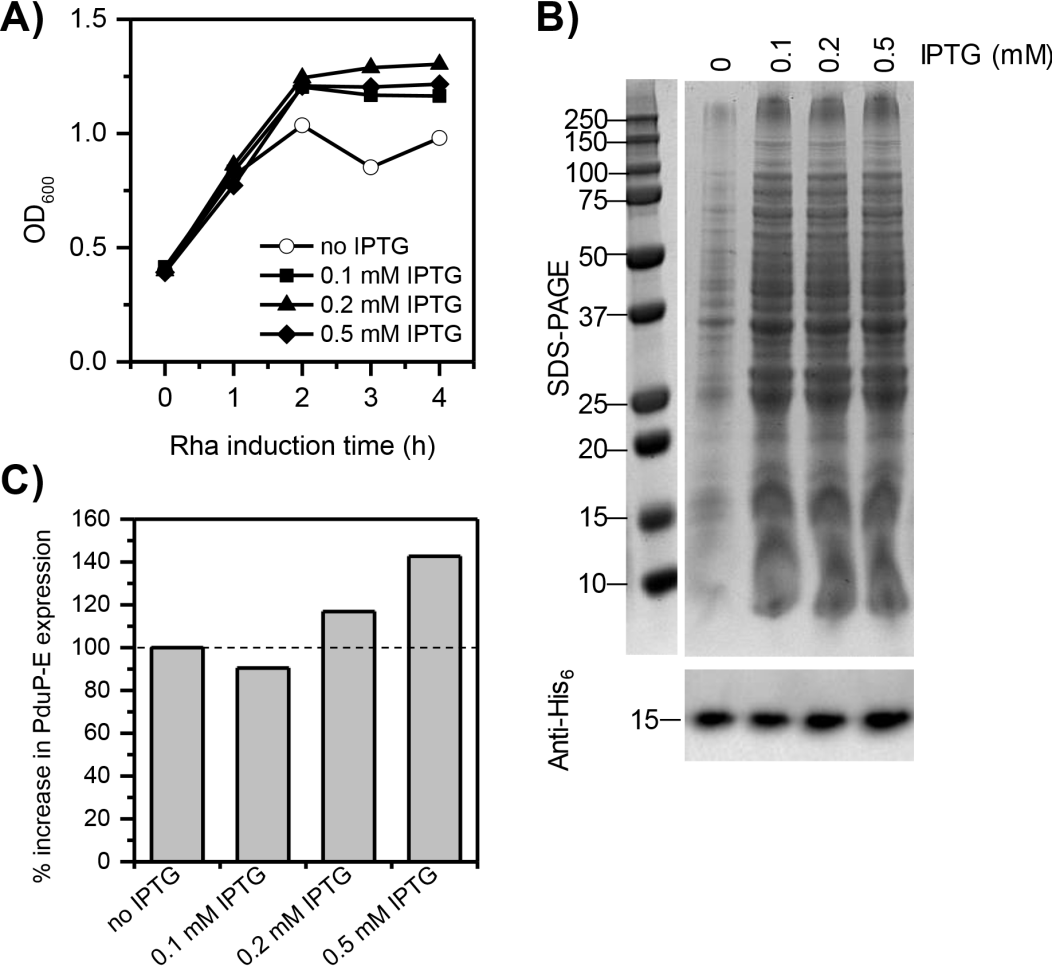


**Figure S4. Effect of different IPTG concentrations on expression of PduP-E.** (A) Growth curves for Ec3087 under different IPTG inductions concentrations. All cultures were induced with 0.1 mM rhamnose at t=0. IPTG was added at t=-3 h. No IPTG (○); 0.1 mM IPTG (■); 0.2 mM IPTG (▲); 0.5 mM IPTG (♦). (B) SDS-PAGE and anti-His_6_ Western blot at 4 h post-rhamnose induction. The equivalent of 50 µL and 0.8 µL of original culture were analyzed by SDS-PAGE and Western blot, respectively. Sizes of molecular weight standards in kDa are shown on the left. (C) Relative increase in PduP-E expression compared to the “no IPTG” condition based on Western blot intensities.


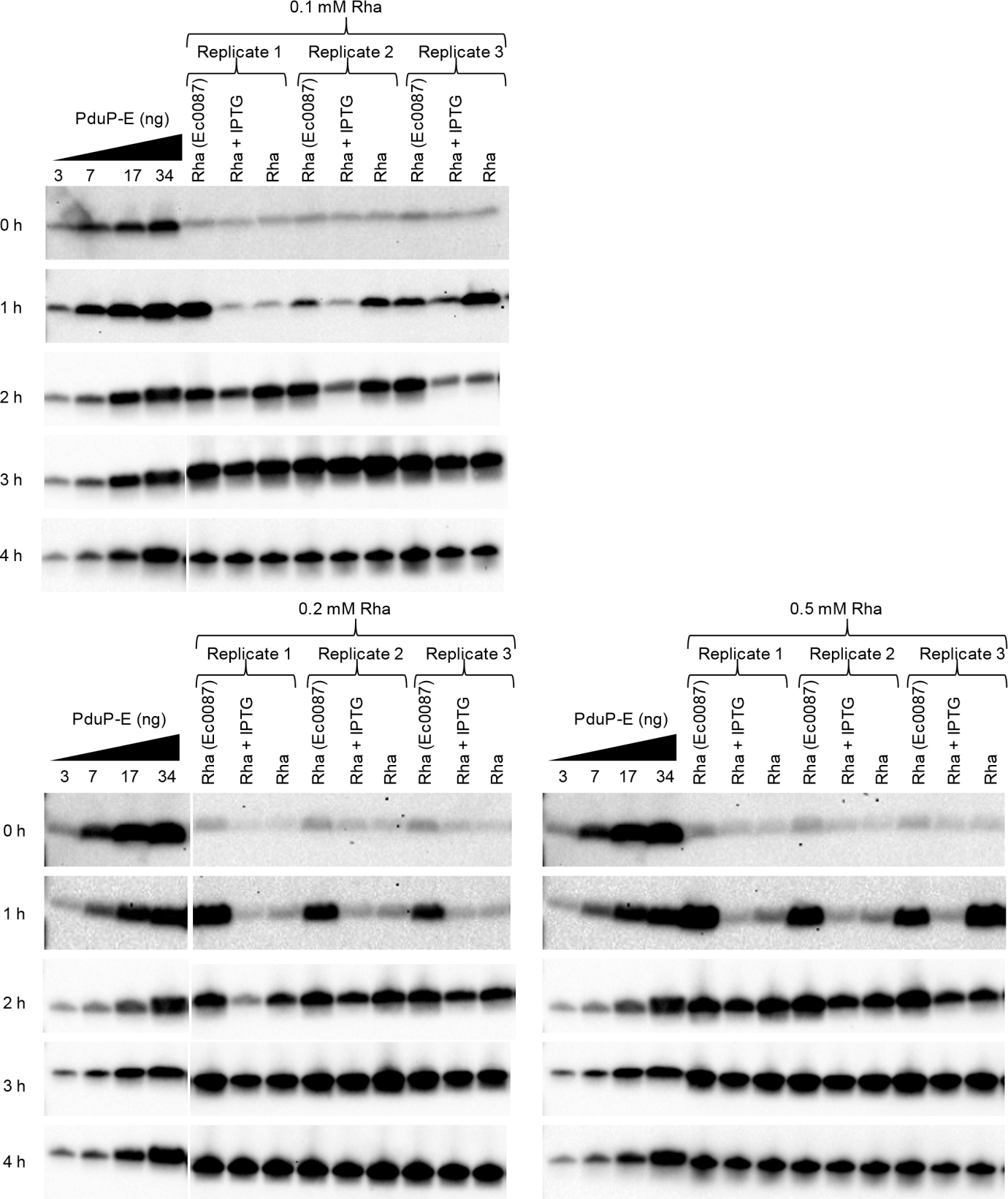


**Figure S5. Raw anti-His_6_ Western blot data represented in Figure 2.** Samples were normalized to equivalent µL*OD_600_ (approximation for equal number of cells). Samples were loaded so that they fell within the PduP-E standard curve. Standard curves and adjacent samples were loaded on the same Western blot. For all 0 h and 1 h samples, 14 µL of OD_600_=4 suspension was loaded. For all 2 h samples and the 3 h samples under 0.1 mM Rha, 8 µL of OD_600_=0.8 suspension was loaded. For the 3 h samples under 0.2 and 0.5 mM Rha and the 4 h samples under 0.2 mM Rha, 4 µL of OD_600_=0.8 suspension was loaded. For the 4 h samples under 0.1 and 0.5 mM Rha, 4 µL of OD_600_=0.4 suspension was loaded.


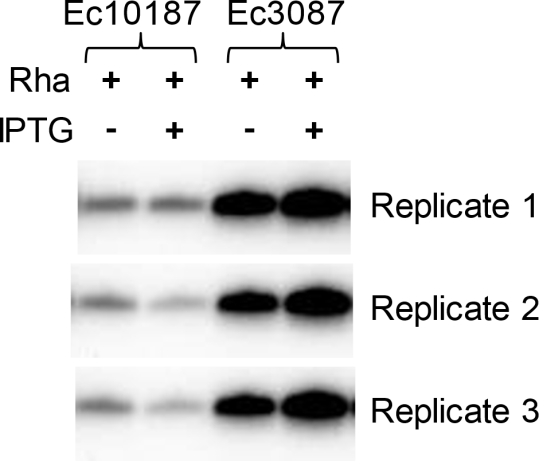


**Figure S6. Raw anti-His_6_ Western blot data represented in Figure 3D.** Each replicate set was performed on the same Western blot. Intensities were determined and normalized to Ec3087, Rha only. Average relative intensities are presented in Figure 3D.


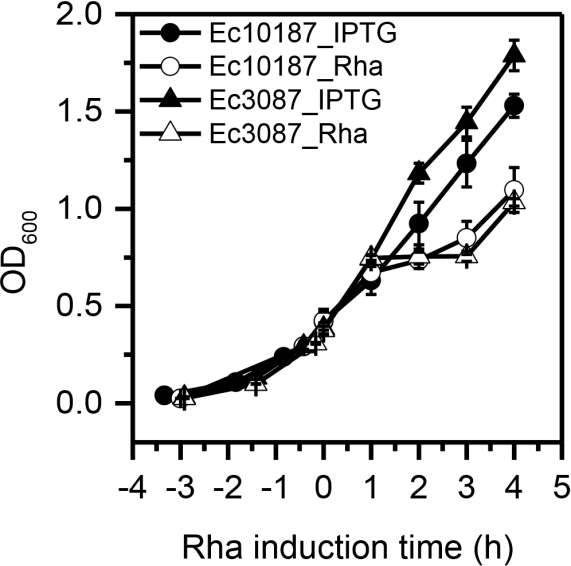


**Figure S7. Growth curves for Ec10187 and Ec3087 under IPTG only and rhamnose only induction.** IPTG was added at t=-3 h and rhamnose was added at t=0. Error bars represent data from 3 biological replicates. Ec10187 under IPTG only (●); Ec10187 under rhamnose only (○); Ec3087 under IPTG only (▲); Ec3087 under rhamnose only (∆). Similar toxicity is observed with Ec10187 and Ec3087 induced with rhamnose only.


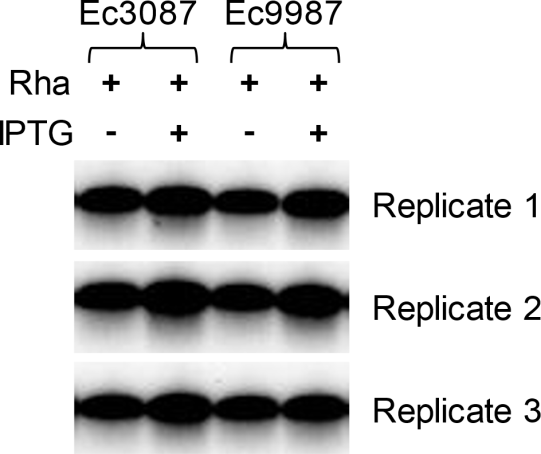


**Figure S8. Raw anti-His_6_ Western blot data represented in Figure 4D.** Each replicate set was performed on the same Western blot. Intensities were determined and normalized to Ec3087, Rha only. Average relative intensities are presented in Figure 4D.


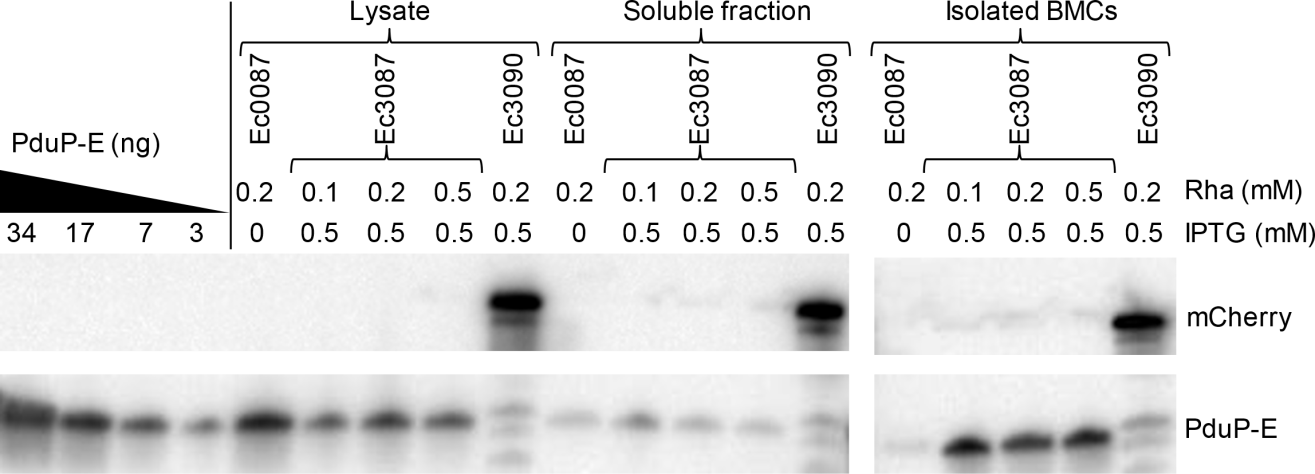


| Strain | Induction conditions | Fraction | Loaded  His protein (ng)*^a^* | Equivalent volume loaded (µL)*^b^* | Total volume (µL)*^c^* | Total His protein (µg)*^d^* | % in total lysate*^e^* |
| --- | --- | --- | --- | --- | --- | --- | --- |
| Ec0087  (PduP-LysE) | 0.2 mM Rha | Lysate | 18 | 0.054 | 3,800 | 1,300 | 100% |
|  |  | Soluble | 1.2 | 0.063 | 3,800 | 75 | 5.8% |
|  |  | BMC | 1.0 | 6.5 | 400 | 0.063 | 0.0049% |
| Ec3087  (PduP-LysE+full Pdu) | 0.1 mM Rha + 0.5 mM IPTG | Lysate | 6.9 | 0.021 | 4,100 | 1,300 | 100% |
|  |  | Soluble | 2.6 | 0.028 | 4,100 | 380 | 28% |
|  |  | BMC | 14 | 0.31 | 420 | 18 | 1.4% |
| Ec3087  (PduP-LysE+full Pdu) | 0.2 mM Rha + 0.5 mM IPTG | Lysate | 10 | 0.028 | 4,500 | 1,700 | 100% |
|  |  | Soluble | 1.3 | 0.035 | 4,500 | 170 | 10% |
|  |  | BMC | 12 | 0.57 | 420 | 8.7 | 0.52% |
| Ec3087  (PduP-LysE+full Pdu) | 0.5 mM Rha + 0.5 mM IPTG | Lysate | 7.7 | 0.027 | 4,000 | 1,100 | 100% |
|  |  | Soluble | 1.1 | 0.037 | 4,000 | 120 | 11% |
|  |  | BMC | 13 | 0.45 | 420 | 12 | 1.1% |
| Ec3090  (PduP-mCherry+full Pdu) | 0.2 mM Rha + 0.5 mM IPTG | Lysate | 100 | 0.025 | 7,000 | 29,000 | 100% |
|  |  | Soluble | 90 | 0.029 | 7,000 | 22,000 | 75% |
|  |  | BMC | 82 | 0.47 | 420 | 73 | 0.25% |

*^a^* Amount of His_6_-tagged PduP-E or PduP-mCherry in the above Western blot as determined by the PduP-E standard curve. The anti-His_6_ is assumed to detect an equal number of PduP-E and PduP-mCherry molecules (*i.e.*, 1 ng of PduP-E (14.8 kDa) detected is equivalent to 2.1 ng of PduP-mCherry (31.2 kDa) detected).

*^b^* Equivalent volume of the undiluted sample that was loaded on the Western blot. Undiluted samples were diluted and appropriate volumes (5-15 µL) were used to load 1 µg of protein (based on Bradford assay quantitation).

*^c^* Total volume of the undiluted sample from 200 mL of culture.

*^d^* Calculated total amount of His_6_-tagged PduP-E or PduP-mCherry found in the undiluted sample.

*^e^* Percent of His_6_-tagged protein in the sample relative to in the total lysate.

**Figure S9. Raw anti-His_6_ Western blot data for amounts presented in Table 2.** Figure above, Western blot. Cells induced under the indicated rhamnose (Rha) and IPTG conditions were collected at 3 h post-rhamnose induction for BMC isolation. Lysate is the sample immediately after French press lysis. Soluble fraction is the sample after the initial 12,000 x *g* centrifugation step. Isolated BMCs is the final purified BMC suspension. Standard curve of known PduP-E amounts is shown on the left. 1 µg of each sample was loaded on the Western blot gel based on Bradford assay quantitation. Table below, quantitation of the amounts of PduP-E and PduP-mCherry during isolation of BMCs.


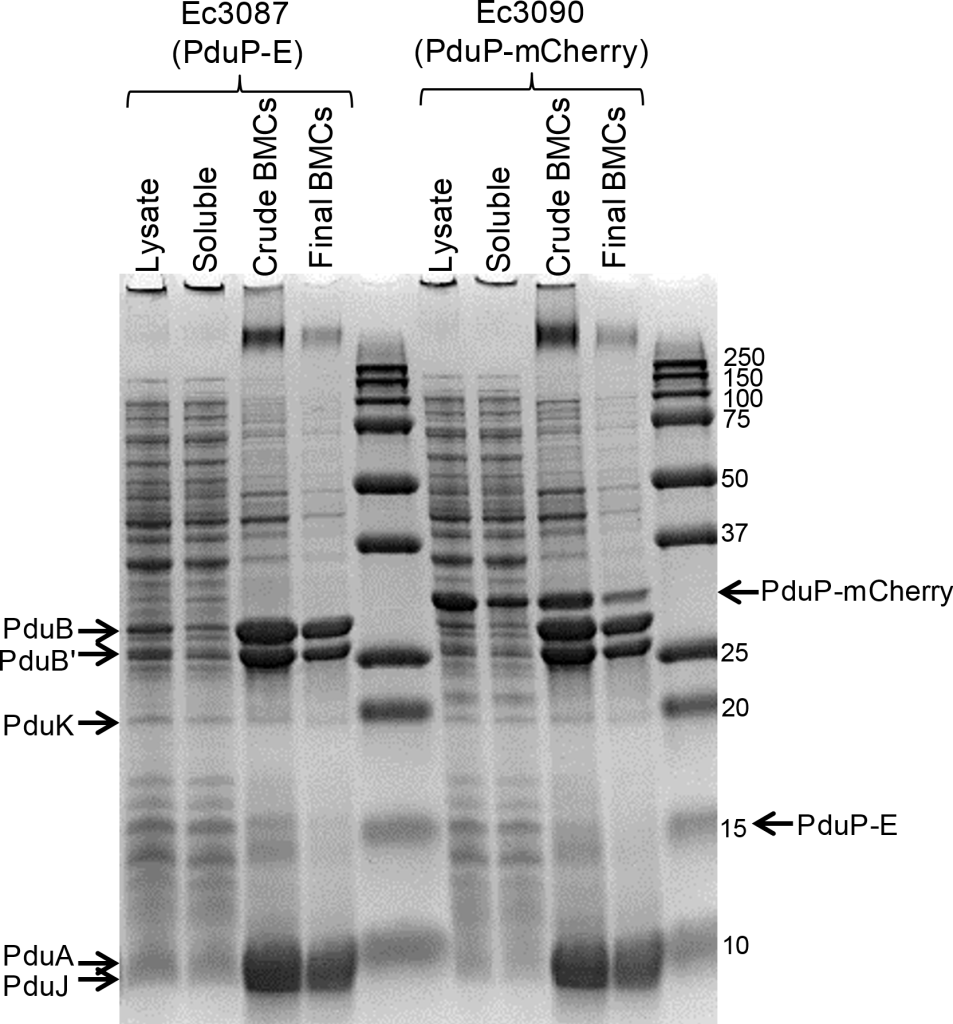


**Figure S10. SDS-PAGE analysis of samples during isolation of BMCs.** BMCs were isolated from Ec3087 and Ec3090 cells induced with 0.5 mM IPTG and 0.2 mM rhamnose for 3 h. Lysate, 23-27 µg of sample immediately after French Press lysis. Soluble, 20 µg of sample after the first 12,000 x *g* centrifugation step. Crude BMCs, 20 µg of sample after re-suspension in buffer B. Final BMCs, 12 µg of sample after the final three centrifugation steps at 12,000 x *g*. Equal volumes of lysate and soluble samples were loaded to observe the loss of BMC shell proteins from the first centrifugation step. Locations of PduP-mCherry, PduP-E, PduA, PduB, PduB’, PduJ, and PduK are shown above. Location of molecular weight standards are denoted in kDa on the right.


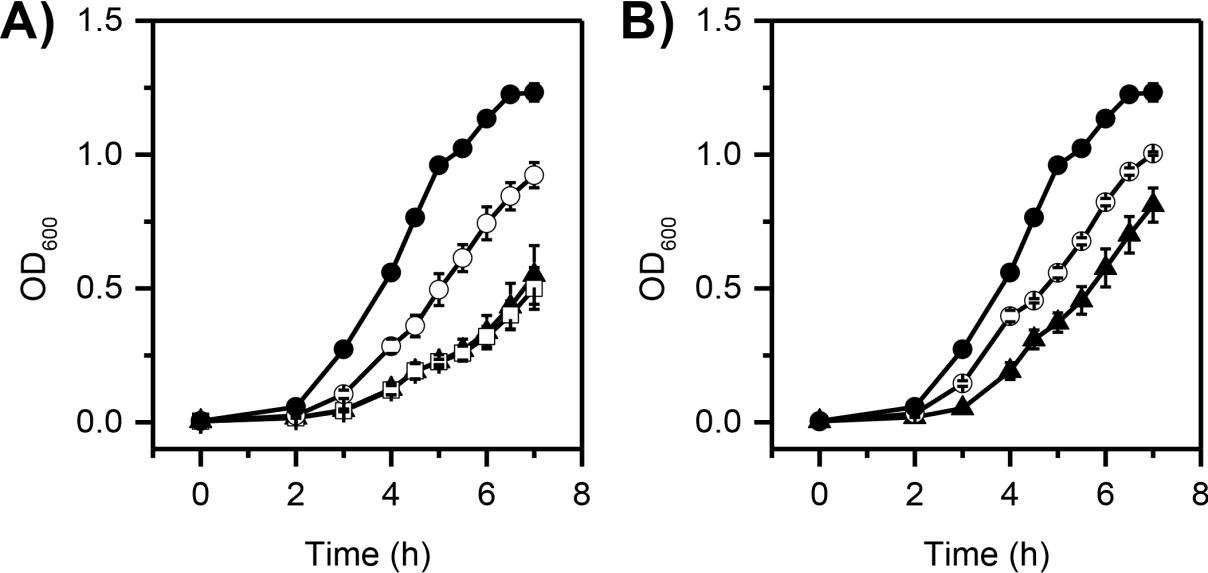


**Figure S11. *B. lincheniformis* growth inhibition assays for PduP-E (A) and non-tagged E (B).** *B. lincheniformis* cells were grown in 100 µL of LB in a 96-well plate and growths were monitored by OD_600_. PduP-E and non-tagged E were added to 0.5 µM. Equivalent volumes of the controls were added. (A) Normal growth (●); EBB control (○); PduP-E purified from Ec3087 cells induced with rhamnose and IPTG (▲); PduP-E purified from Ec3087 cells induced with rhamnose only (□). (B) Normal growth (●); Factor Xa in EBB control (○); non-tagged E after Factor Xa proteolysis.
